# Supplementary material for: Beyond blast: enabling microbiologists to better extract literature, taxonomic distributions and gene neighbourhood information for protein families
Source: Microb Genom. 2024 Feb 7;10(2):001183. doi: 10.1099/mgen.0.001183 (PMC10926702; doi:10.1099/mgen.0.001183)
Supplement: Supplementary material 1 [file mgen-10-1183-s001.pdf]

Supplemental Materials

Index

**Headings for each section of supplementary content are hyperlinked to facilitate document navigation.*

Supplementary Data

S1. All keywords accumulated for DUF34 protein family during the comprehensive published data capture process.
S2. Curated sets of tools organized by: (a) orthology-/homology-limited and phyletic patterning tools; (b) gene neighborhood; and (c) synteny tools.
S3. All curated yields of specialized corpus search tools: PubMed, EuropePMC, PubTator, Scinapse.
S4. Simple Comparison of Text-based vs. Sequence-based Publication Retrieval.
S5. Publication Retrieval Method Comparison — Single-sequence vs. HMM-based PaperBLAST Methods.
S6. Venn Diagram Data for the Comparison of the Two Major PaperBLAST Retrieval Methods.
S7. Publication Retrieval Method Comparison — Single-sequence vs. HMM-based vs. Idealized "QCC" Cycle Method.
S8. Venn Diagram Data Comparing PaperBLAST Retrieval Methods to "QCC Cycle" Approach.
S9. Comparison of the PaperBLAST Results of Several, Disparately Related DUF34 Homolog Sequences.
S10. Final Summary Venn Diagram of all Methods' Results Relevant to DUF34.

Supplementary Tables

S1. All 65 gene families characterized by the Laboratory of Valérie de Crécy-Lagard, PhD.
S2. Results of investigation comparing sequence-based search tool PaperBLAST (two methods) to those of the idealized “QCC” method.

Supplementary Elaborations

S1. Explanation of XynX “productive ignorance” example.

Supplementary Figures

S1. Seed Information Assessment: establish understanding of starting information in preparation for the capture of published data.
S2. Retrieve sequences using known names/aliases.
S3. Family-level analyses.
S4. Example of over-propagation of root family attribution in EggNOG Database as a result of fusions.
S5. WorldWideScience.org-, ScienceResearch.com-generated diagrams of keywords.
S6. Coincidental homonyms are challenges for text-based search engines, even those as specialized as PubTator.
S7. FlaGs example output.
S8. EFI-GNT example output.
S9. Annotree example: single family, PF02591, in bacteria.
S10. COGNAT example output.
S11. SubtiWiki (CoreWiki) beta feature of the database’s Genomic Neighborhood Comparison viewer of the DUF34 homolog of B. subtilis, YqfO (BSU_25170).
S12. Example tree output of MicrobesOnline.
S13. Screengrab demonstrating different “filters” for target family recognition, particularly used differentially with multiple targets (MicrobesOnline).
S14. Screengrab demonstrating navigation to family-specific trees in MicrobesOnline via the “Gene Info” tab of each entry.
S15. “Families” (COGs) in STRING search tool options.
S16. KO identifiers being supplied to KEGG orthology tool (multiple families, limited genome customization).
S17. Example CAGECAT output using DUF34 and COG1579 homolog sequences: MSMEG_4307 (NCBI-ProteinID: ABK73705); MSMEG_4306 (NCBI-ProteinID: ABK70599).
S18. Example output for FunCoup using “YbgI” of *E. coli*.
S19. BV-BRC protein family sorter, general output example (a) with additional viewer examples.

S20. BV-BRC protein family sorter output, Families.
S21. EggNOG Phylogenetic Profile tool example output using COG0327, COG3323, and COG1579.
S22. KBase workflow for generating a “gene tree” (as regarded by KBase) from several select genomes.
S23. Genomic Context Visualizer example output.
S24. GizmoGene example output.

Supplementary References

Supplementary Data

**Supplementary data files in submission packet are also accessible via the accompanying FigShare.*

Data S1. All keywords accumulated for DUF34 protein family during the comprehensive published data capture process.

Data S2. Curated sets of tools organized by: (a) orthology-/homology-limited and phyletic patterning tools; (b) gene neighborhood; and (c) synteny tools.

Data S3. All curated yields of specialized corpus search tools: PubMed, EuropePMC, PubTator, Scinapse. Each search engine was queried using the 10 selected keywords thought to represent the most commonly associated names/aliases associated with the target protein family (i.e., “NGG1 interacting factor 3”, “NIF3”, “NIF3L1”, “GTP cyclohydrolase 1 type 2”, “DUF34”, “YbgI”, “PF01784”, “COG0327”, “YqfO”, and “COG3323”). Respective yields were exported from each engine’s results and, subsequently, manually reviewed for relevance rated at three levels: 1) “Focal”, a true positive hit in which the target homolog was mentioned in either the title and/or the abstract; 2) “Non-focal”, a true positive hit in which the target homolog, while not mentioned in the title or abstract, was mentioned in another subsection or supplemental data of the publication; and 3) “False Positive”, a curated and confirmed false positive hit in which the target homolog was falsely identified among any of the yielded publication’s text. These three ratings constitute the three left-most columns of each subset of exported hits per search engine (respective publication’s row assigned a “yes” in the column corresponding to one of the three rating categories for which it was found affirmative, reflecting the curation results or relevance status). Tables reflecting the curation summaries of each raw export (i.e., each keyword/query per search engine) were generated to document curation process (see “Export Subsets, Curation”). The total number of raw yields varied per search engine due to the variable productivity of individual engines and select keywords within those search engines. Raw yields (i.e., returned publications) for each keyword per search engine were also included in this supplementary dataset, accompanying the export summaries; queries returning no hits were unable to be exported and, therefore, are not represented within a raw file (see export summaries for which queries resulted in no hits, and, therein, which query yields were left out of the raw data files; Data S3 table includes a key for all raw export files). The results of this survey are illustrated in **Figure 4** of the main text.

Supplementary Tables

Table S1. All 65 gene families characterized by the Laboratory of Valérie de Crécy-Lagard, PhD. (Asterisks indicate homologs in human)

Pathway	Name	UniProt Representative	COG/DUF/PFAM	PMID
**RNA Modification**				
Agmitidine Synthase	TiaS	O59476	COG1571	18844986; 20139989
Archaeosine Synthesis	QueF-like	A3MSP1	COG0780 QueF-paralog	22032275; 28383498
Archaeosine Synthesis	Gat/QueC fusion	Q981C9	COG0449/COG0603	22032275
Archaeosine Synthase	ArcS	Q58428	COG1549, DUF5591, TGT paralog	20129918
5-methylaminomethyl-2-thiouridine synthase	MnmC1/C2 fusion	P77182	COG4121/COG0579	17673083
Pre-Q_0 Reductase (Queuosine Biosynthesis	QueF	Q46920	COG2904/0780	14660578; 15767583
Queuosine/Archaeosine Biosynthesis	QueC	O31675	COG0603	14660578
Queuosine/Archaeosine Biosynthesis	QueD	O31676	COG0720	14660578
Queuosine/Archaeosine Biosynthesis	QueE	O31677	COG0602	14660578
Queuosine Biosynthesis	QueH	Q9WZJ0	DUF208	28128549
PreQ0/preQ1 transport	YhhQ	P37619	COG1738	28208705
Queuosine (Q) hydrolase	CD1682/QueK	Q186N9	COG1957 paralog	31481610
Queuine lyase	CD1684/QueL	Q186P0	COG1244 paralog	31481610
Q transport	CT140	O84142	COG1738 paralog	31481610
Q Synthesis	CT193	O84196	COG0343 paralog	31481610
Queuosine Salvage	Qng1*	Q9HDZ9	DUF2419	24911101; 36610787
tRNA-ac4C Biosynthesis	TmcA	D4GW73	COG1444	19478918
tRNA-m1A22 Biosynthesis	TrmK	P54471	COG2384	18420655; 17852564
tRNA-m1Psi54 synthesis	PusY	D4GTL8	COG1901/DUF358	22274953;
tRNA-t6A37 Synthesis (Universal)	TsaC *	P45748	COG0009	19287007
tRNA-t6A37 Synthesis (Universal)	TsaC2, Sua5	P32579	PF03481	19287007
tRNA-t6A37 Synthesis (Bacteria)	TsaB	O05516	COG1214	21285948
tRNA-t6A37 Synthesis (Bacteria)	TsaD	P05852	COG0533	21285948
tRNA-t6A37 Synthesis (Bacteria)	TsaE	P0AF67	COG0802	22378793
tRNA-t6A37 Synthesis (Eukarya/Archaea Cyto)	Kae1*	P36132	PF00814	21285948
tRNA Dihydrouridine Synthase (Bacteria)	DusABC*	P32695	COG0042	11983710
Wybutosine Biosynthesis	TYW1, WyeA*	Q08960	PF00258/PF04055/PF08608	16162496
Wyosine Derivative Biosynthesis (Archaea)	Taw22	Q9V2G1	COG2520	20382657
tRNA Psi 32/32 Synthase (*B. subtilis*)	YjbO	O31613	COG0564 paralog	32629984
rRNA Psi Sythase (*B. subtilis*)	YhcT	P54604	COG0564 paralog	32629984
tRNA m6t6A	TrmO*	P28634	COG1720	25063302
**DNA Modification**				
dADG modification	DpdA	A0A3A3JBR7	COG0343 TGT paralog	30159947
dADG modification	DpdB	A0A3A3NHD9	pfam14072 DndB paralog	30159947
dADG modification	DpdC	A0A3A3IHU3	DUF308	30159947
**Small Molecule/Cofactor Metabolism**				

Folate Synthesis (Bacteria, FolB Shunt)	PTPS-III	A0A098MZ39	COG0720 paralog	19395485
Folate FolC-like (Chlamydiae)	CT611	O84617	COG1478	17645794
GTP Cyclohydrolase I type 2	FolE2	P94398	COG1469	17032654
Folate, New PABA Synthesis	CT610	O84616	COG5424	25006229
Iron-sulfur Cluster Repair	YgfZ*	P0ADE8	COG0354, GcvT paralog	20489182
Pterin Biosynthesis (Archaea)	FolB2/MptD	Q57851	COG2098/DUF372/381	22931285
Pterin Biosynthesis (Archaea)	FolK2/MptE	Q59028	COG1634/DUF115	22931285
Tetrahydromonapterin Synthesis	FolX	P0AC19	COG1539	19897652
Tetrahydromonapterin Synthesis	FolM	P0AFS3	COG1028 paralog	19897652
Thiamine Metabolism	TenAE	Q9ASY9	COG0812	25014715
Zinc Homeostasis	YeiR*	P33030	COG0523 paralog	15690043
R,S SAM hydrolase	Sare_1364	A8M783	DUF62	32776704
Oxoproline Metabolism	YbgJ	P0AAV4	COG20149	28830929
Oxoproline Metabolism	YbjK	P75745	COG1984	28830929
Oxoproline Metabolism	YbgL	P75746	COG1540 paralog	28830929
Riboflavin	YbiA/RibX	P30176	COG3236	25431972
Thiamine Metabolism	At4g29530	Q9SU92	PF06888 paralog	26537753
Thiamine Metabolism (Plants)	TenAC	F4KFT7	IPR036412/IPR016084 /IPR004305	27677881
Phosphopanteine Hydrolase	At4g32180*	Q8L5Y9	DUF89_II (PanK fusion)	27322068
B12 Metabolism	PF0295	Q8U404	COG2103/DUF71 paralog	23013770
PLP Homeostasis	YggS/PLPHP/PLPBP*	P67080	COG0325	26872910
**Protein Modification**				
Beta-Lysylation of Elongation Factor P	YjeA	P0A8N7	COG226 paralogs	20070887; 21841797
Beta-Lysylation of Elongation Factor P	YjeK	P39280	COG0535 paralogs	20070887; 21841797
Diphthamide Biosynthesis	Dph6*	Q12429	COG2103/DUF71 paralog	23013770
Deoxyhypusine Biosynthesis	HVO_2299	D4GWG4	COG0010 paralogs	28053595
**Carbon Source Catabolism**				
Mannitol-phosphate Dehydrogenase/Phosphatase	MtlD	Q6FBP5	PF13419/PF08125	24800891
Sugar-phosphate and Nucleotide Hydrolase	PH1575	O59272	DUF89_I	27322068
Sugar-phosphate Hydrolase	YMR027W	Q04371	DUF89_III	27322068
3-Oxo-tertronate Kinase	YgbK	Q46889	DUF1537 paralog	27402745; 27294475
D-Threonate Kinase	DtnK	Q8ZRS5	DUF1537 paralog	27402745; 27294475
D-Threonate 4-phosphate Dehydrogenase	PdxA2	P58718	COG1995 paralog	27402745; 27294475
**Unpublished (papers in-prep)**				
nm5U34 Synthase	YtqA	O35008	COG1242	
nm5U34 Methylase	YtqB	O34614	COG2519	
dPreQ1 Methylase	DpdM	M4PNV1	PF03692 (paralog?)	https://biorxiv.org/cgi/content/short/20

				23.04.13.536721v1
dPreQ1 Formyltransferase	DpdN	NA, ncbi: YP_010114479.1	PF00551 paralog	https://biorxiv.org/cgi/content/short/2023.04.13.536721v1
CDG Decarboxylase	DpdL	M4SNA7	COG0720 paralog	https://biorxiv.org/cgi/content/short/2023.04.13.536721v1
$preQ_0/preQ_1$ Transporter	Bifidobacterium breve CREST homolog	A0A0M3T8W5	PF03006/COG1272 paralog	
$preQ_0/preQ_1$ Transporter	*Bartonella henselae* MFS homolog	WP_011180872.1	PF07690/COG2814 paralog	
$preQ_0/preQ_1/q$	*Acidobacteria bacterium* DMT homolog	A0A2V9U0M9	PF07168 paralog	

Table S2. Results of investigation comparing sequence-based search tool PaperBLAST (two methods) to those of the idealized "QCC" method. "Focal" and "False Positive" publication labels have been bolded in the table.

	**Method**			
	**PaperBLAST**			**QCC**
	**HMM**	**Single Sequence/UniProt ID**		
**Relevance**	**PF01784**	***H. sapiens* (Q9GZT8)**	***E. coli* (P0AFP6)**	
Non-Focal				Ahmed 2011
**Focal**	Akiyama 2003	Akiyama 2003		Akiyama 2003
Non-Focal				Alalouf 2011
Non-Focal	Alam 2011	Alam 2011	Alam 2011	Alam 2011
Non-Focal	Anderson 2010	Anderson 2010		
Non-Focal				Alderman 2019
Non-Focal				Alqazlan 2020 (thesis)
Non-Focal				Amon 2010 (thesis)
Non-Focal				Antelmann 2005
Non-Focal				Antoniali 2017
Non-Focal	Araújo 2020	Araújo 2020		Araújo 2020
Non-Focal				Ashburner 1999
Non-Focal				Aurass 2009
Non-Focal				Avican 2021
**Focal**				Bagautdinov 2008
Non-Focal				Bai 2017
Non-Focal				Bashir 2020
Non-Focal				Baysal 2013
Non-Focal	Belvin 2019	Belvin 2019		Belvin 2019
Non-Focal				Bermudez 2015
Non-Focal				Bhasin 2008
Non-Focal				Bhatraju 2015
Non-Focal	Bleichert 2020	Bleichert 2020		Bleichert 2020
Non-Focal				Boot 2016
Non-Focal				Bootsma 2010 (patent)
Non-Focal				Boswell 2018
Non-Focal				Breker 2014
Non-Focal				Brosnahan 2019
Non-Focal				Brosnahan 2020 (thesis)
**False Positive**	Brady 1989		Brady 1989	
Non-Focal				Bruce 2012 (patent)

Non-Focal				Burnstein 2016
Non-Focal	Byrne 2014		Byrne 2014	Byrne 2014
Non-Focal				Camp 2016
Non-Focal				Cardenas 2009
Non-Focal				Chang 2016
Non-Focal				Charlton 2015
Non-Focal				Chauhan 2015
Non-Focal				Chen 1999
**Focal**				Chen 2010a
Non-Focal				Chen 2010b
**Focal**	Chen 2014	Chen 2014	Chen 2014	Chen 2014
Non-Focal				Cherkas 2016
Non-Focal				Chiesa 2020
**Focal**	Choi 2013	Choi 2013		Choi 2013
Non-Focal				Chung 2013
Non-Focal				Codrich 2019
Non-Focal				Comas 2008
Non-Focal				Conn 2017
**Focal**				Constantine 2009
Non-Focal	Cox 2020		Cox 2020	
Non-Focal	Crapoulet 2006	Crapoulet 2006		Crapoulet 2006
Non-Focal				Cury 2020
Non-Focal	Czubat 2020	Czubat 2020		
Non-Focal	Da Costa 2018	Da Costa 2018		Da Costa 2018
Non-Focal				Daas 2016
Non-Focal				Daas 2018
Non-Focal				Dalia 2011 (thesis?)
Non-Focal				Damianou 2020
Non-Focal	Danelishvili 2005	Danelishvili 2005		Danelishvili 2005
Non-Focal				Daou 2019
Non-Focal				Dapas 2019 (thesis)
Non-Focal				Dartigalongue 2001
Non-Focal				Degnan 2005
Non-Focal				DeLoney 2002
Non-Focal	De Sordi 2015	De Sordi 2015		
Non-Focal	Díaz-Mejía 2009		Díaz-Mejía 2009	Díaz-Mejía 2009
Non-Focal				Dickey 2013 (thesis)
Non-Focal				Ding 2015
Non-Focal				Dionisio 2016
Non-Focal				Ditse 2017
Non-Focal	Dong 2008	Dong 2008		Dong 2008
Non-Focal				Drummelsmith 2007
Non-Focal				Ducret 2021
Non-Focal				Dudin 2017
Non-Focal				Duncan 2017
Non-Focal				Dunman 2001
Non-Focal				Duzyj 2014
Non-Focal				Edwards 2013
Non-Focal				El Bounkari 2009
Non-Focal	Esser 2008		Esser 2008	Esser 2008
Non-Focal				Facciuolo 2013
Non-Focal				Facciuolo 2016
Non-Focal				Falkenberg 2019
Non-Focal				Fang 2010
Non-Focal	Fenner 2019	Fenner 2019		Fenner 2019
Non-Focal				Fontes 2018 (thesis)
Non-Focal				Freiberg 2016
**Focal**	Fujishiro 2014	Fujishiro 2014	Fujishiro 2014	Fujishiro 2014
Non-Focal				Fujishiro 2015

Non-Focal				Fujishiro 2016
Non-Focal				Fukushima 2020
Non-Focal	Furuta 2010		Furuta 2010	Furuta 2010
Non-Focal				Galperin 2010
Non-Focal	Gangaiah 2013		Gangaiah 2013	
Non-Focal	Gangaiah 2014		Gangaiah 2014	Gangaiah 2014
Non-Focal				Gao 2019
Non-Focal	Gaudet 2011		Gaudet 2011	
Non-Focal	Gawinecka 2012	Gawinecka 2012		Gawinecka 2012
Non-Focal				Geislet 1992
Non-Focal				Geyer 2019
Non-Focal	Ghaemmaghami 2003	Ghaemmaghami 2003		Ghaemmaghami 2003
Non-Focal				Ghersi 2011
Non-Focal				Giannangelo 2018 (thesis)
Non-Focal				Gifford 2000
Non-Focal				Giotti 2019
Non-Focal				Giovannelli 2017
Non-Focal				Girinathan 2018
Non-Focal				Giuffrida 2006
**Focal**	Godsey 2007	Godsey 2007	Godsey 2007	Godsey 2007
Non-Focal				Gooderham 2008 (thesis)
Non-Focal				Graf 2018
Non-Focal	Gupta 2017	Gupta 2017		Gupta 2017
Non-Focal				Gupta 2018
Focal				Hadano 2001
Non-Focal				Hadj-Hamou 2010 (thesis)
Non-Focal	Han 2006		Han 2006	Han 2006
Non-Focal	Happonen 2019	Happonen 2019	Happonen 2019	Happonen 2019
Non-Focal				Harrison 2021
Non-Focal				Hart 2018
Non-Focal				Hayles 2013
Non-Focal				He 2019
Non-Focal	Hendrickson 2010	Hendrickson 2010		
Non-Focal				Hermans 2008 (patent)
Non-Focal				Hews 2019
Non-Focal				Hladik 2019
Non-Focal	Ho 2015	Ho 2015		Ho 2015
Non-Focal				Ho 2016
Non-Focal				Hossain 2021
Non-Focal	Huang 2015	Huang 2015	Huang 2015	
Non-Focal				Huang 2019
Non-Focal				Huston 2017 (lecture)
Non-Focal				Huttlin 2010
Non-Focal				Iyer 2016
Non-Focal				Jackson 2013
Non-Focal				Jama 2014 (thesis)
Non-Focal				Jiang 2013
Non-Focal	Jiang 2023	Jiang 2023	Jiang 2023	
Non-Focal				Johnson 2018
Non-Focal				Johnson 2019 (preprint)
Non-Focal				Jones 2006
Non-Focal				Jostes 2019 (thesis)
Non-Focal	Joshi 2020		Joshi 2020	
Non-Focal				Jothi 2006
Non-Focal				Kai 2016
Non-Focal				Kalari 2013
**Focal**	Kanesaki 2021	Kanesaki 2021		
**Focal**				Karniely 2006
Non-Focal	Kawabata 2019	Kawabata 2019		

Non-Focal				Kaysser 2010
Non-Focal				Kerns 2020 (thesis)
Non-Focal				Kim 2018
Non-Focal	Kölbel 2019	Kölbel 2019		Kölbel 2019
Non-Focal	Kolker 2004		Kolker 2004	Kolker 2004
**Focal**	Kuan 2013	Kuan 2013	Kuan 2013	Kuan 2013
Non-Focal				Kumar 2018
Non-Focal				Kusonmano 2018
Non-Focal				Kwon 2005
Non-Focal	Labandeira-Rey 2009		Labandeira-Rey 2009	Labandeira-Rey 2009
**Focal**	Ladner 2003		Ladner 2003	Ladner 2003
**Focal**				Lamba 2013 (poster abstract)
Non-Focal	Lasserre 2006			
Non-Focal	Leang 2005	Leang 2005	Leang 2005	Leang 2005
Non-Focal				Lee 2008 (thesis)
Non-Focal	Li 2010	Li 2010	Li 2010	Li 2010
Non-Focal	Li 2017	Li 2017		Li 2017
Non-Focal	Liang 2013	Liang 2013	Liang 2013	Liang 2013
Non-Focal				Lie 2013
Non-Focal				Lin 2004
Non-Focal				Lin 2019
Non-Focal				Liu 2013
Non-Focal				Liu 2014
Non-Focal				Liu 2019
Non-Focal				Lively 2010
Non-Focal	Livengood 2008		Livengood 2008	Livengood 2008
Non-Focal				Long 2020
Non-Focal				López-Madrigal 2013
Non-Focal				Lu 2015
Non-Focal				Luo 2005 (thesis)
Non-Focal				Luo 2007
Non-Focal				Lv 2021
Non-Focal				Mahdi 2013
Non-Focal				Makarova 2010
Non-Focal				Malan-Müller 2020
**Focal**				Malik 2020 (preprint)
Non-Focal	Malik-Kale 2008	Malik-Kale 2008	Malik-Kale 2008	Malik-Kale 2008
**Focal**	Manan 2018	Manan 2018	Manan 2018	Manan 2018
Non-Focal				Manzano-Marin 2019
Non-Focal	Manzourolajdad 2015	Manzourolajdad 2015		Manzourolajdad 2015
**Focal**	Martens 1996	Martens 1996		Martens 1996
Non-Focal				Martin 2002
Non-Focal				Martin 2018
Non-Focal				Martinot 2018
Non-Focal				Massart 2015
Non-Focal				McBrearty 2019 (thesis)
Non-Focal				McKee 2018
Non-Focal				McKenzie 2012
Non-Focal	McKenzie 2015	McKenzie 2015		McKenzie 2015
**False Positive**	McLeod 2017	McLeod 2017		
Non-Focal	Medrano 2009		Medrano 2009	Medrano 2009
Non-Focal				Meibom 2004
Non-Focal	Melian 2021	Melian 2021	Melian 2021	
**Focal**				Merla 2004
Non-Focal				Meshram 2019
Non-Focal				Minias 2015
Non-Focal				Minias 2018
Non-Focal				Moreira 2008
Non-Focal	Moreno 2018	Moreno 2018		Moreno 2018

Non-Focal				Morgenstern 2017
Non-Focal				Munoz 2020
Non-Focal				Nalls 2009
Non-Focal	Naqvi 2015		Naqvi 2015	Naqvi 2015
Non-Focal	Naqvi 2018		Naqvi 2018	
Non-Focal				Nelson 2007
Non-Focal	Neville 2020	Neville 2020	Neville 2020	
Non-Focal				Niehaus 2015
Non-Focal				Nowlan 2020
Non-Focal				Nowlan 2021
Non-Focal				O'Connor 2012
Non-Focal				Ogura 2010
Non-Focal				Ogura 2016
Non-Focal				Ogura 2018
**Focal**				Ogura 2019
Non-Focal				O'Hanlon 2011
Non-Focal				Oja 2018
Non-Focal	Page 2005	Page 2005		Page 2005
Non-Focal				Pan 2012
Non-Focal	Parente 2016	Parente 2016		Parente 2016
Non-Focal				Park 2007
Non-Focal				Passalacqua 2012
Non-Focal				Patel 2015
Non-Focal				Pattrick 2019
Non-Focal	Paul 2017	Paul 2017		Paul 2017
Non-Focal				Peng 2019
Non-Focal	Pereira 2005	Pereira 2005		
Non-Focal	Pereira 2011	Pereira 2011		Pereira 2011
Non-Focal				Perez-Samper 2018
Non-Focal				Phuphisut 2018
Non-Focal	Pompesiello 2001		Pompesiello 2001	Pompesiello 2001
Non-Focal	Porcelli 2013	Porcelli 2013	Porcelli 2013	Porcelli 2013
Non-Focal				Prahlad 2020
Non-Focal	Prunetti 2016		Prunetti 2016	Prunetti 2016
**False Positive**	Prusty 2013		Prusty 2013	
Non-Focal				Pulido 2020 (thesis)
Non-Focal				Punekar 2016
**Focal**				Qijing 2007 (translated)
Non-Focal				Qiu 2019
Non-Focal				Qu 2020
Non-Focal				Quigley 2014
Non-Focal				Rachel 2012
Non-Focal				Rahmani-Badi 2016
Non-Focal				Rajathei 2013
Non-Focal				Raman 2006
Non-Focal				Ramaniuk 2018
Non-Focal				Rankin 2002
**Focal**	Reed 2021	Reed 2021	Reed 2021	Reed 2021
Non-Focal	Reinders 2006	Reinders 2006		Reinders 2006
Non-Focal				Resch 2016
Non-Focal				Rijlaarsdam 2016
Non-Focal				Risler 2012
Non-Focal				Rollins 2006
Non-Focal	Rooney 2009		Rooney 2009	Rooney 2009
Non-Focal	Rooney 2011	Rooney 2011	Rooney 2011	Rooney 2011
Non-Focal				Rosenbaum 2011
Non-Focal				Ryan 2005
Non-Focal				Rylander 2011
Non-Focal				Saez 2019

**Focal**	Saikatendu 2006	Saikatendu 2006		Saikatendu 2006
Non-Focal				Sailani 2015
Non-Focal	Sainsbury 2012		Sainsbury 2012	Sainsbury 2012
Non-Focal				Samper 2019
Non-Focal				Samudrala 2009
Non-Focal				Sarker 2012
Non-Focal				Schlenker 2011 (thesis)
Non-Focal				Schneeweiss 2020
Non-Focal				Schrader 2016
Non-Focal				Selby 2017
Non-Focal				Selim 2021
**Focal**	Sergeeva 2018		Sergeeva 2018	Sergeeva 2018
Non-Focal	Shahbaaz 2013		Shahbaaz 2013	Shahbaaz 2013
Non-Focal				Shakya 2020 (preprint)
Non-Focal				Shimada 2011
Non-Focal				Shin 2011
Non-Focal				Shulami 1999
Non-Focal				Shulami 2007
**Focal**	Shulami 2014		Shulami 2014	Shulami 2014
Non-Focal				Sigdel 2011
Non-Focal				Simon 2016
Non-Focal				Simonis 2012
Non-Focal				Simpson 2015
Non-Focal				Skottman 2005
Non-Focal				Skunca 2013
Non-Focal	Spinola 2010		Spinola 2010	Spinola 2010
Non-Focal	Stabler 2010			Stabler 2010
Non-Focal				Starkey 2009
Non-Focal				Stenger 2019 (thesis)
Non-Focal				Stern 2020
Non-Focal				Steyn 2017 (thesis)
Non-Focal				Strillacci 2020
Non-Focal				Su 2012
Non-Focal				Suzuki 2006
Non-Focal				Ta 2010
Non-Focal				Tajkarimi 2017
**Focal**	Tascou 2000	Tascou 2000		Tascou 2000
**Focal**	Tascou 2003	Tascou 2003		Tascou 2003
Non-Focal				Thankam 2018
Non-Focal				Thorenoor 2010
Non-Focal				Tian 2014
Non-Focal				Tian 2020
**Focal**	Tomoike 2009		Tomoike 2009	Tomoike 2009
Non-Focal				Tran 2018
Non-Focal				Tsuiko 2016
Non-Focal				Turlin 2006
Non-Focal				Uxa 2019
Non-Focal	van Diemen 2017	van Diemen 2017		van Diemen 2017
Non-Focal	Van Dyke 2016		Van Dyke 2016	Van Dyke 2016
Non-Focal				van Ouwerkerk 2019
Non-Focal				Volha 2019 (thesis)
Non-Focal			Wagley 2014	Wagley 2014
Non-Focal				Waldor 2013
Non-Focal				Wang 2010
**False Positive**	Wang 2012	Wang 2012		Wang 2012
Non-Focal				Wang 2015
Non-Focal				Wang 2018
Non-Focal				Wang 2021
Non-Focal				Watkins 2008

Non-Focal				Watkins 2010
Non-Focal				Wei 2001
Non-Focal	Wei 2016	Wei 2016		Wei 2016
Non-Focal				Weinzierl 2008
Non-Focal				Wilkinson 2018 (thesis)
Non-Focal	Williams 2013	Williams 2013		Williams 2013
Non-Focal				Willis 2010 (thesis)
Non-Focal				Winer 2011
Non-Focal				Wittchen 2018
Non-Focal				Wu 2019
Non-Focal				Xi 2011
Non-Focal				Xia 2017
Non-Focal				Xiang 2012
**Focal**				Xie 2019
Non-Focal	Xu 2021	Xu 2021		
Non-Focal				Yan 2014
Non-Focal				Yan 2020
Non-Focal				Yang 2013
Non-Focal				Yao 2016
**Focal**				Yu 2015
Non-Focal				Yu 2018
Non-Focal				Yuan 2006
Non-Focal				Zamanian-Daryoush 2020
Non-Focal				Zhao 2018
Non-Focal				Zheng 2020
Non-Focal				Zuccotti 2008

Supplementary Elaborations

Elaboration S1. Explanation of XynX "productive ignorance" example.

XynX, a DUF34 homolog of *Geobacillus stearothermophilus* T-6, was identified and described by a couple researcher groups over the course of several papers between the years 1994 and 2016 [1–6]. Upon identification, because of its genomic location and position within an operon of biosynthetic interest, it was named with respect to neighboring genes. This naming and system-level characterization were completed almost entirely independent of the preceding literature of the protein family of which it was a recognizable member. Additionally, and although the genomic information of the organism had been purportedly annotated and respective data made publicly available, it was—upon review of Reed et al. *Biomolecules* 2021 [7]—discovered that the genome and related annotation information was not as accessible as had been claimed. It had been observed that the complete genome and any annotated, encoded proteins had been intermittently submitted and updated by the submitter over the course of 15 years (1993-2008). Although the EuropePMC Data subsection for the earliest publication [1] lists 51 links to individual UniProt entries and a single ENA link to 57 nucleotide records (accessed May 20, 2022; https://europepmc.org/article/MED/8031084): the original publisher does not provide links to this information (now hosted at the website for the parent journal, ASM; https://doi.org/10.1128/aem.60.6.1889-1896.1994); this paper does not provide any systematic accession identifiers for the data allegedly being reported (i.e., the genome); and only 56 of the 57 EuropePMC reported genes are actually listed in the ENA database under the queryable publication-associated ID, 8031084.

It is hypothesized that this EuropePMC data being reported as being associated with this publication [1] is mistakenly attributed to this particular record. At this time, it is suspected that the accessioned data referenced in many of these online publications has been retroactively linked to these other works from the Gat et al. 1994 record and, subsequently, the Shulami et al. 2011 publication in the Journal of Bacteriology [5] (https://doi.org/10.1128/JB.00222-11; first cited by Alalouf et al. 2011 in the Journal of Biological Chemistry (PMID: 21994937) [4], methods section) with which a GenBank record was submitted for the 77,747 bp (linear) of the *G. stearothermophilus* T-6 genome that was reported to contain "the xylan, xylose, arabinan, and arabinose utilization region" relevant to the study. The record, GenBank: DQ868502.2, is titled "Geobacillus stearothermophilus strain T-6 genomic sequence", suggesting that the genome is completely sequenced; GenBank record is dated "26-JUL-2016". This record replaced the earlier GenBank record, DQ868502.1, titled "Geobacillus stearothermophilus strain T-6 xylan utilization gene cluster, complete sequence; and NAD(P)H-dependent flavin oxidoreductase (orfD) gene, partial cds" dated 01-FEB-2007. As per Gat et al., 1994, the earliest genomic record (annotated with only a few CDSs, but largely unannotated) was submitted to GenBank, DDBJ, and EMBL (ENA) with the accession identifier Z29080. The first in-text description of *xynX* and its encoded protein of the same name was in Shulami et al., *Applied and Environmental Microbiology* 2007 [3] and described as a "xylan utilization gene". In Alalouf et al., 2011 [4], XynX is described as "a putative regulatory gene with unknown function". It's putative cooperative regulation of *xynA* expression alongside XylR and CodY is described in more detail in Shulami et al., *Journal of Biological Chemistry* 2014 [5]. No description of the family to which XynX belongs is mentioned, no attempt to identify the associated protein family is made, despite assigning the protein a name and describing a functional association. Although these characterizations (and alias) were assigned with ignorance of the protein family, the functional association of XynX as a DUF34 member was not dissimilar from subsequent characterizations, for example, in *Bacillus subtillis* [8], and, therefore, cannot be discounted in its contributions to describing functional associations at the family level.

Supplementary Figures

Figure S1. Seed Information Assessment: establish understanding of starting information in preparation for the capture of published data.

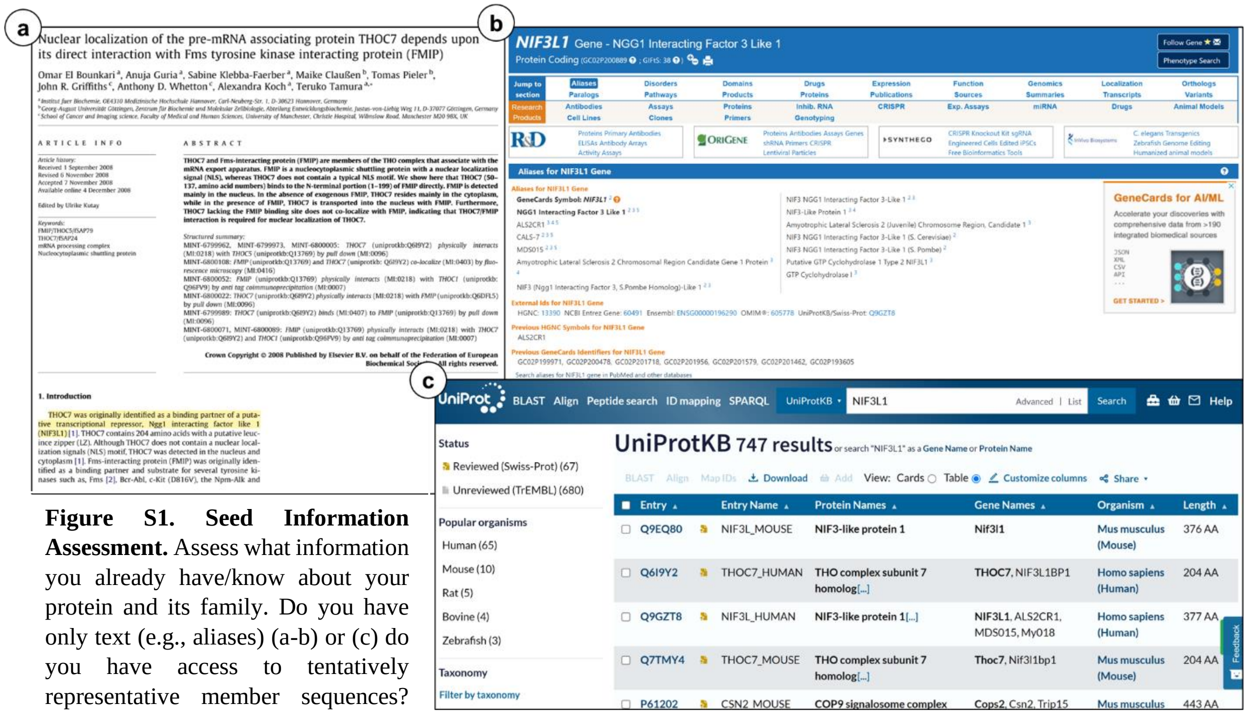

Figure S1. Seed Information Assessment. Assess what information you already have/know about your protein and its family. Do you have only text (e.g., aliases) (a-b) or (c) do you have access to tentatively representative member sequences? Both (a-c) and, if so, how many? Do you suspect that they are adequately representative of the family?

Figure S2. Retrieve sequences using known names/aliases.

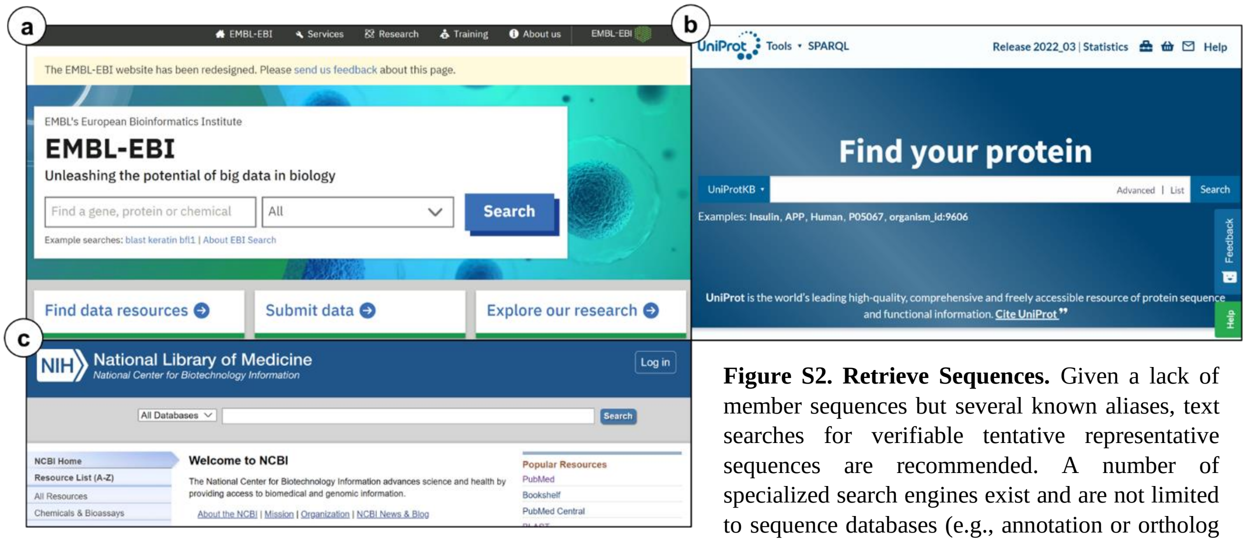

Figure S2. Retrieve Sequences. Given a lack of member sequences but several known aliases, text searches for verifiable tentative representative sequences are recommended. A number of specialized search engines exist and are not limited to sequence databases (e.g., annotation or ortholog databases). Here, examples include (a) EMBL-EBI broad search, (b) UniProtKB, and (c) NIH National Library of Medicine all-database search.

Figure S3a. Family-level analysis examples from Annotree (left) and EFI Taxonomy (right).

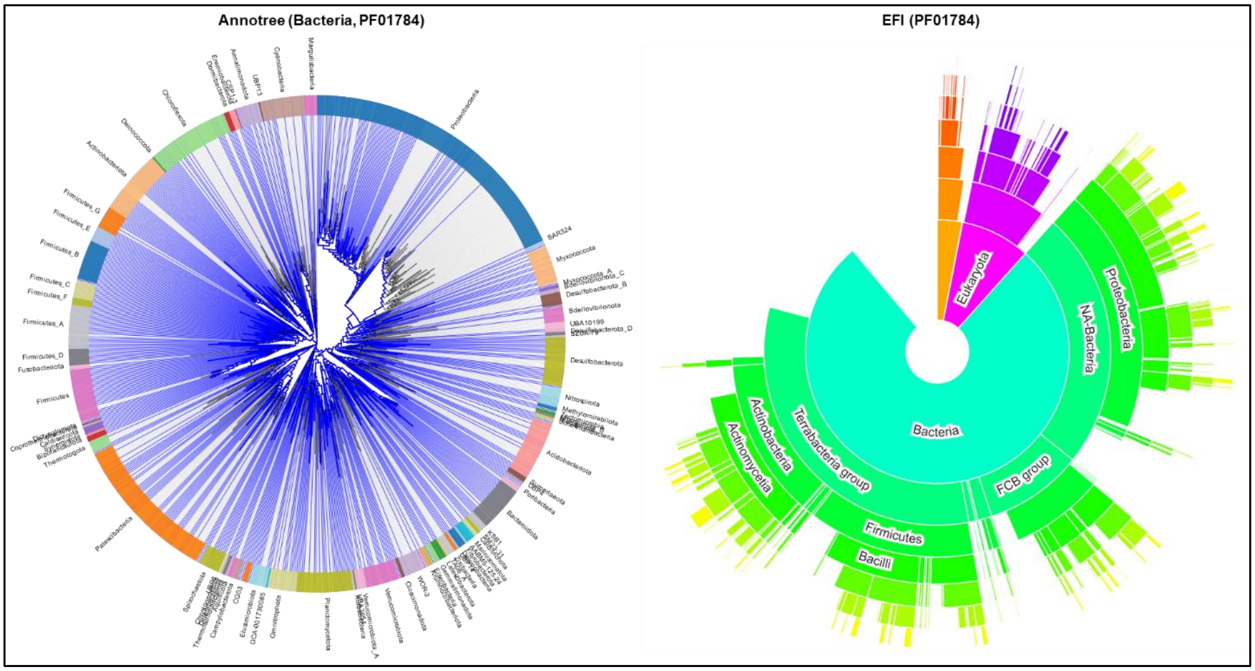

Figure S3b. Family-level analyses using various protein family classification databases (i.e., Pfam, CDD, OrthoMCL, InterPro, OrthoInspector, EggNOG, ENSEMBL tools), of which can be used to approximate architectural subgroups in concert with using representative sequences as inputs into the sequence-based search tools (e.g., i.e., PaperBLAST) for corpus retrieval.

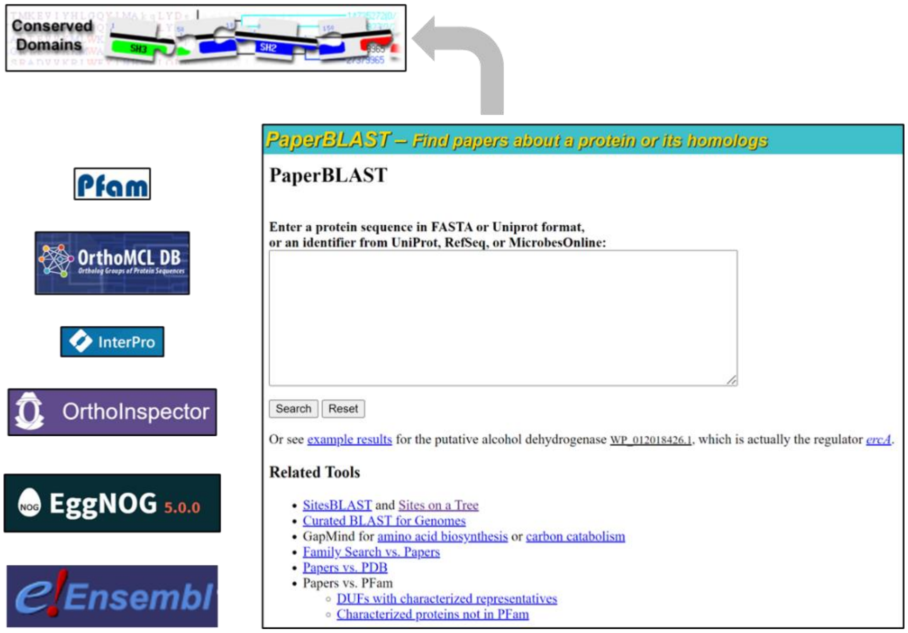

Figure S4. Example of over-propagation of root family attribution in EggNOG Database as a result of fusions. EggNOG aggregates data from other resources and often these collections accrue family members that are domain architectural variants and contain non-family sequence annotations. These annotations are not filtered out or flagged in any way and require the user to discern these erroneous aggregates on their own. (a) EggNOG root cluster of the DUF34 protein family. Added notation of screenshot: red bars underline the incorrect annotations aggregated from KEGG, one biologically incorrect based on published data, a second resulting from a fusion sequence of COG0327 and COG1579 [PMID: 34572495], and a third that is a product of a gene fusion of a eukaryotic DUF34 homolog and CIAO (COG2319). (b) Link-out to the first of three incorrect KO entries listed EggNOG listed KO, K22391, for the enzyme activity of GTP cyclohydrolase I; KO entry lists the corresponding COG, though in this case, no additional COGs are aggregated in KEGG Orthology. (c) Link-out to the second of three listed incorrect KOs of DUF34, that for an uncharacterized bacterial protein family, COG1579 (K07164), an annotation recognized as being associated with the DUF34 family by EggNOG—an association that is likely through the known fusion with the *Wollinella succinogenes* DUF34 homolog [PMID: 34572495] that may or may not have functionally diverged—while KO does not identify the source of this association. (d) Link-out to the third of three incorrect KOs listed in the entry card for LCOG0327 in EggNOG, CIAO (COG2319), also a product of fusion sequences within the DUF34 family. (e) COG0327 (child of root, LCOG0327) "Duplications profile" view.

a.

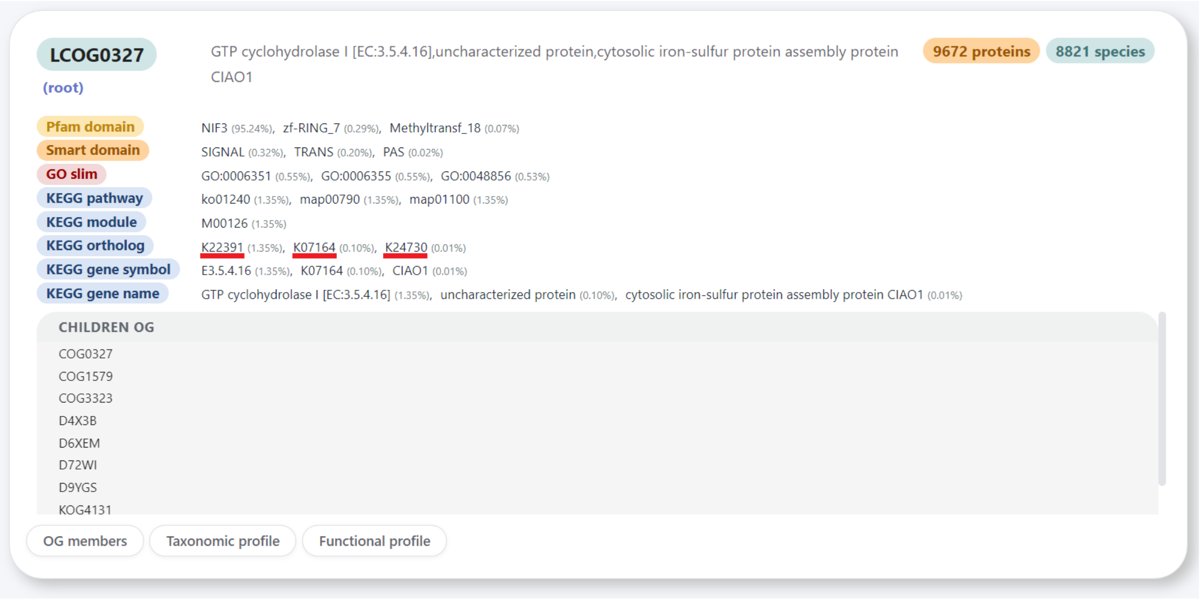

b.

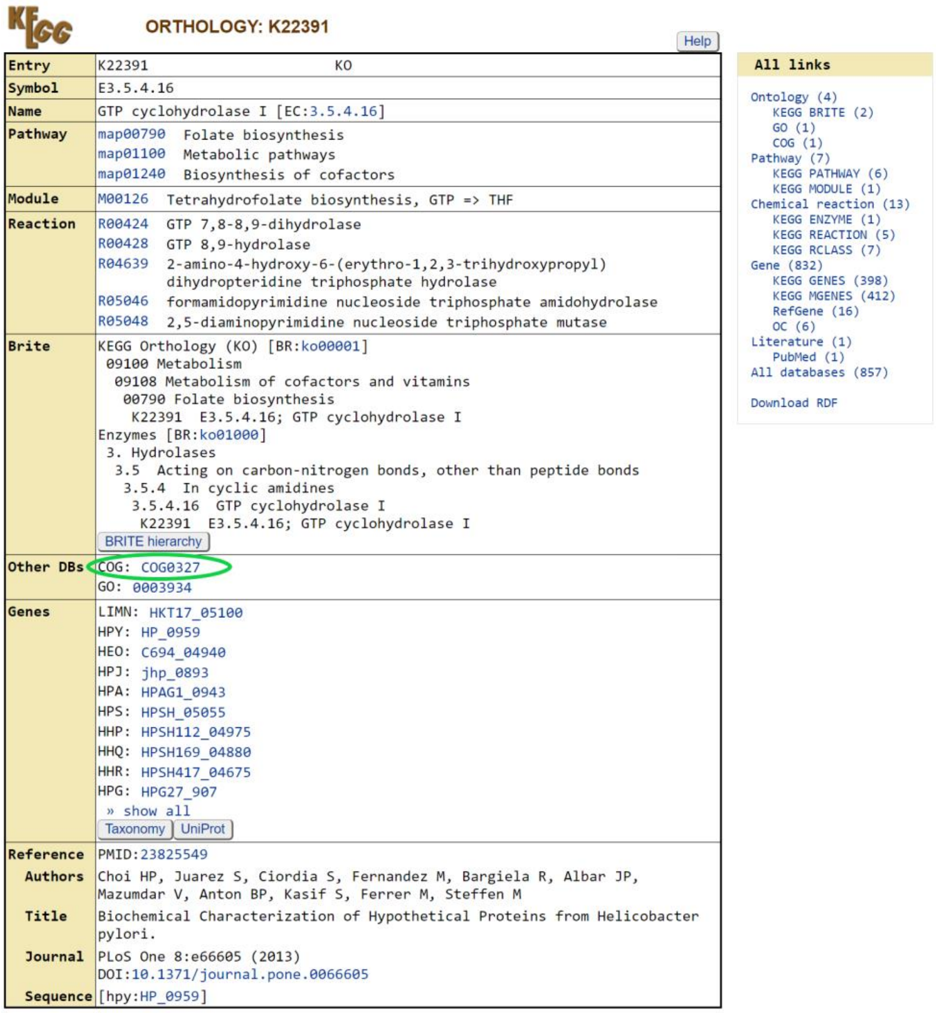

ORTHOLOGY: K22391

Entry	K22391 KO
Symbol	E3.5.4.16
Name	GTP cyclohydrolase I [EC:3.5.4.16]
Pathway	map00790 Folate biosynthesis<br>map01100 Metabolic pathways<br>map01240 Biosynthesis of cofactors
Module	M00126 Tetrahydrofolate biosynthesis, GTP => THF
Reaction	R00424 GTP 7,8-8,9-dihydrolase<br>R00428 GTP 8,9-hydrolase<br>R04639 2-amino-4-hydroxy-6-(erythro-1,2,3-trihydroxypropyl) dihydropteridine triphosphate hydrolase<br>R05046 formamidopyrimidine nucleoside triphosphate amidohydrolase<br>R05048 2,5-diaminopyrimidine nucleoside triphosphate mutase
Brite	KEGG Orthology (KO) [BR:ko00001]<br>09100 Metabolism<br>09108 Metabolism of cofactors and vitamins<br>00790 Folate biosynthesis<br>K22391 E3.5.4.16; GTP cyclohydrolase I<br>Enzymes [BR:ko01000]<br>3. Hydrolases<br>3.5 Acting on carbon-nitrogen bonds, other than peptide bonds<br>3.5.4 In cyclic amidines<br>3.5.4.16 GTP cyclohydrolase I<br>K22391 E3.5.4.16; GTP cyclohydrolase I<br>BRITE hierarchy
Other DBs	COG: COG0327<br>GO: 0003934
Genes	LIMN: HKT17_05100<br>HPY: HP_0959<br>HEO: C694_04940<br>HPJ: jhp_0893<br>HPA: HPAG1_0943<br>HPS: HPSH_05055<br>HHP: HPSH112_04975<br>HHQ: HPSH169_04880<br>HHR: HPSH417_04675<br>HPG: HPG27_907<br>» show all<br>Taxonomy UniProt
Reference	PMID:23825549
Authors	Choi HP, Juarez S, Ciordia S, Fernandez M, Bargiela R, Albar JP, Mazumdar V, Anton BP, Kasif S, Ferrer M, Steffen M
Title	Biochemical Characterization of Hypothetical Proteins from Helicobacter pylori.
Journal	PLoS One 8:e66605 (2013)<br>DOI:10.1371/journal.pone.0066605
Sequence	[hpy:HP_0959]

Help

All links

Ontology (4)
KEGG BRITE (2)
GO (1)
COG (1)
Pathway (7)
KEGG PATHWAY (6)
KEGG MODULE (1)
Chemical reaction (13)
KEGG ENZYME (1)
KEGG REACTION (5)
KEGG RCLASS (7)
Gene (832)
KEGG GENES (398)
KEGG MGENES (412)
RefGene (16)
OC (6)
Literature (1)
PubMed (1)
All databases (857)

Download RDF

c.

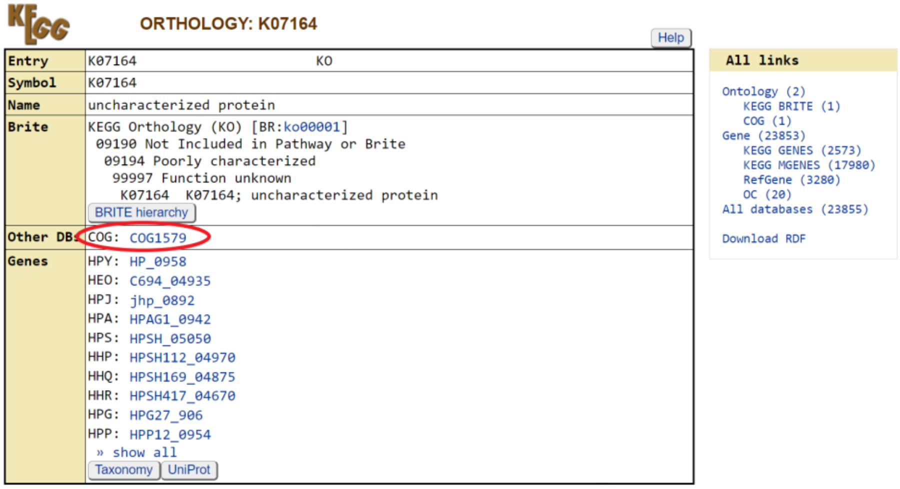

ORTHOLOGY: K07164

Entry	K07164 KO
Symbol	K07164
Name	uncharacterized protein
Brite	KEGG Orthology (KO) [BR:ko00001]<br>09190 Not Included in Pathway or Brite<br>09194 Poorly characterized<br>99997 Function unknown<br>K07164 K07164; uncharacterized protein<br>BRITE hierarchy
Other DBs	COG: COG1579
Genes	HPY: HP_0958<br>HEO: C694_04935<br>HPJ: jhp_0892<br>HPA: HPAG1_0942<br>HPS: HPSH_05050<br>HHP: HPSH112_04970<br>HHQ: HPSH169_04875<br>HHR: HPSH417_04670<br>HPG: HPG27_906<br>HPP: HPP12_0954<br>» show all<br>Taxonomy UniProt

Help

All links

Ontology (2)
KEGG BRITE (1)
COG (1)
Gene (23853)
KEGG GENES (2573)
KEGG MGENES (17980)
RefGene (3280)
OC (20)
All databases (23855)

Download RDF

d.

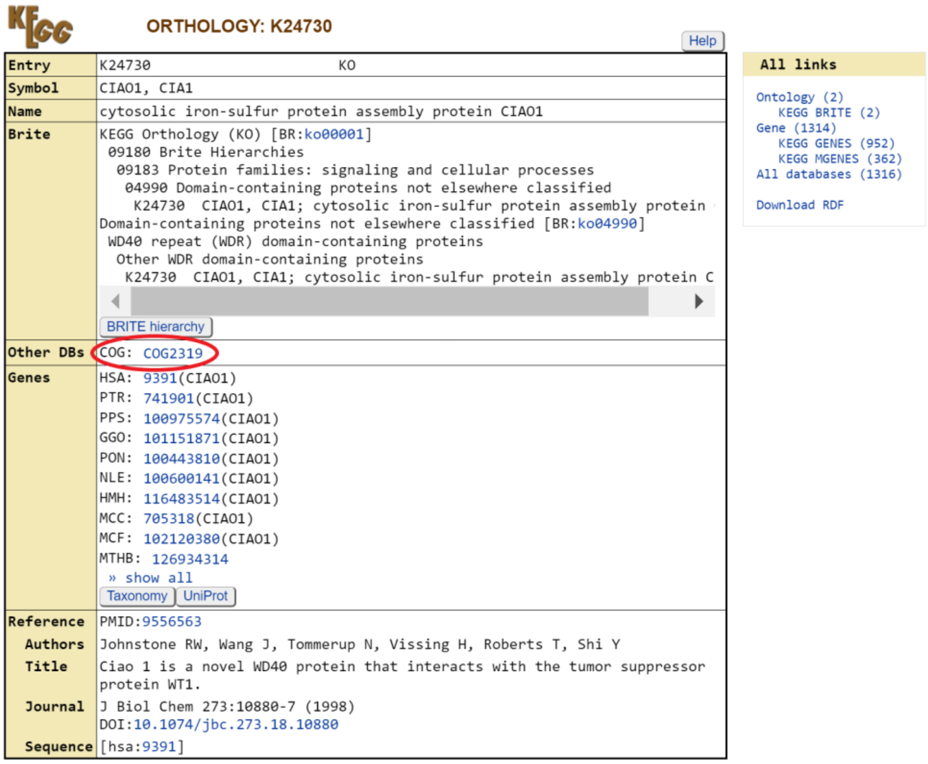
ORTHOLOGY: K24730
Help
Entry K24730 KO
Symbol CIAO1, CIA1
Name cytosolic iron-sulfur protein assembly protein CIAO1
Brite KEGG Orthology (KO) [BR:ko00001]
09180 Brite Hierarchies
09183 Protein families: signaling and cellular processes
04990 Domain-containing proteins not elsewhere classified
K24730 CIAO1, CIA1; cytosolic iron-sulfur protein assembly protein
Domain-containing proteins not elsewhere classified [BR:ko04990]
WD40 repeat (WDR) domain-containing proteins
Other WDR domain-containing proteins
K24730 CIAO1, CIA1; cytosolic iron-sulfur protein assembly protein C
BRITE hierarchy
Other DBs COG: COG2319
Genes HSA: 9391(CIAO1)
PTR: 741901(CIAO1)
PPS: 100975574(CIAO1)
GGO: 101151871(CIAO1)
PON: 100443810(CIAO1)
NLE: 100600141(CIAO1)
HMH: 116483514(CIAO1)
MCC: 705318(CIAO1)
MCF: 102120380(CIAO1)
MTHB: 126934314
» show all
Taxonomy UniProt
Reference PMID:9556563
Authors Johnstone RW, Wang J, Tommerup N, Vissing H, Roberts T, Shi Y
Title Ciao 1 is a novel WD40 protein that interacts with the tumor suppressor protein WT1.
Journal J Biol Chem 273:10880-7 (1998)
DOI:10.1074/jbc.273.18.10880
Sequence [hsa:9391]
All links
Ontology (2)
KEGG BRITE (2)
Gene (1314)
KEGG GENES (952)
KEGG MGENES (362)
All databases (1316)
Download RDF

e.

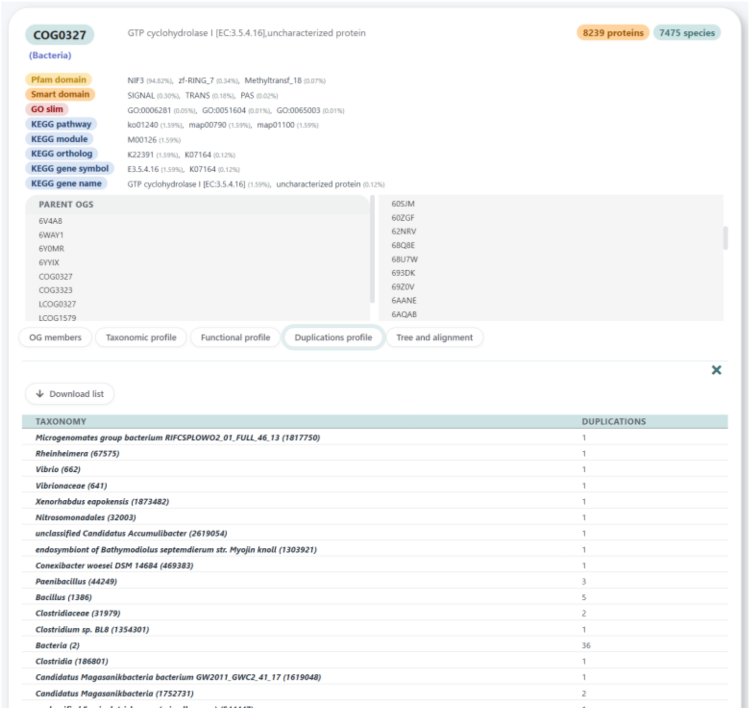
COG0327
(Bacteria)
GTP cyclohydrolase I [EC:3.5.4.16],uncharacterized protein
8239 proteins
7475 species
Pfam domain NIF3 (94.82%), zf-RING_7 (0.34%), Methyltransf_18 (0.07%)
Smart domain SIGNAL (0.30%), TRANS (0.18%), PAS (0.02%)
GO slim GO:0006281 (0.05%), GO:0051604 (0.01%), GO:0065003 (0.01%)
KEGG pathway ko01240 (1.59%), map00790 (1.59%), map01100 (1.59%)
KEGG module M00126 (1.59%)
KEGG ortholog K22391 (1.59%), K07164 (0.12%)
KEGG gene symbol E3.5.4.16 (1.59%), K07164 (0.12%)
KEGG gene name GTP cyclohydrolase I [EC:3.5.4.16] (1.59%), uncharacterized protein (0.12%)
PARENT OGS
6V4A8
6WAY1
6Y0MR
6YYIX
COG0327
COG3323
LCOG0327
LCOG1579
60SJM
60ZGF
62NRV
68Q8E
68U7W
693DK
69Z0V
6AANE
6AQAB
OG members
Taxonomic profile
Functional profile
Duplications profile
Tree and alignment
Download list
TAXONOMY DUPLICATIONS
Microgenomates group bacterium RIFCSPLOWO2_01_FULL_46_13 (1817750) 1
Rheinheimera (67575) 1
Vibrio (662) 1
Vibrionaceae (641) 1
Xenorhabdus eapokensis (1873482) 1
Nitrosomonadales (32003) 1
unclassified Candidatus Accumulibacter (2619054) 1
endosymbiont of Bathymodiolus septemdierum str. Myojin knoll (1303921) 1
Conexibacter woesei DSM 14684 (469383) 1
Paenibacillus (44249) 3
Bacillus (1386) 5
Clostridiaceae (31979) 2
Clostridium sp. BL8 (1354301) 1
Bacteria (2) 36
Clostridia (186801) 1
Candidatus Magasanikbacteria bacterium GW2011_GWC2_41_17 (1619048) 1
Candidatus Magasanikbacteria (1752731) 2

Figure S5. WorldWideScience.org-, ScienceResearch.com-generated diagrams of keywords. Visualized yields illustrate coincidental homonyms of NIF3 (DUF34).

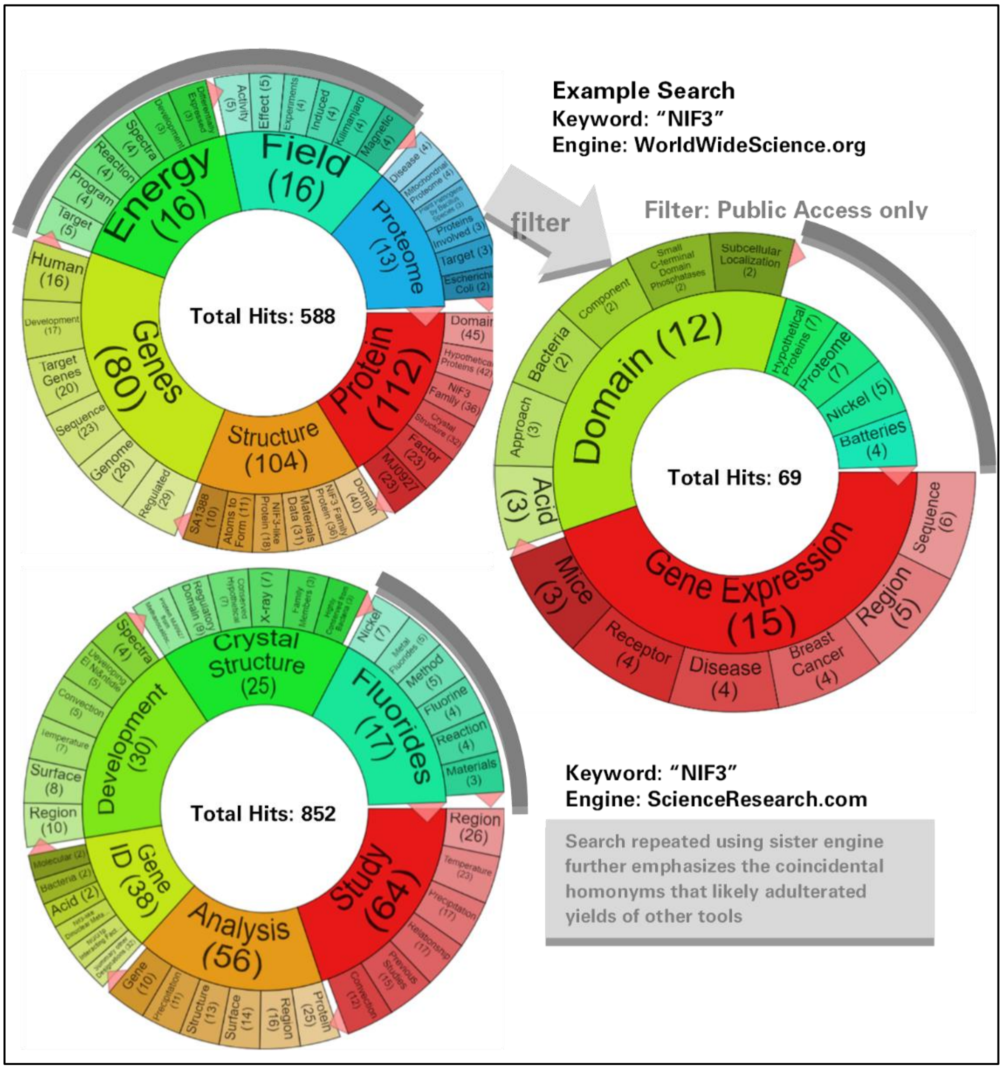

Figure S6. Coincidental homonyms are challenges for text-based search engines, even those as specialized as PubTator. Top screenshot shows PubTator top hits for “GTP cyclohydrolase 1 type 2” search, while the bottom screenshot shows the top hits for alias synonym “GTP cyclohydrolase I type 2”. These searches should hypothetically retrieve identical results; however, in addition to the false synonyms being retrieved due to the automatic generalization of the original search term to “GTP cyclohydrolase”, the engine simultaneously retrieves a correct result in only one of the two case uses (the bottom instance, “GTP cyclohydrolase I type 2”), suggesting that the tool is unable to discern singular “I” usage as the Roman numeral for 1, which is nomenclature practice frequently used in the naming of genes/proteins.

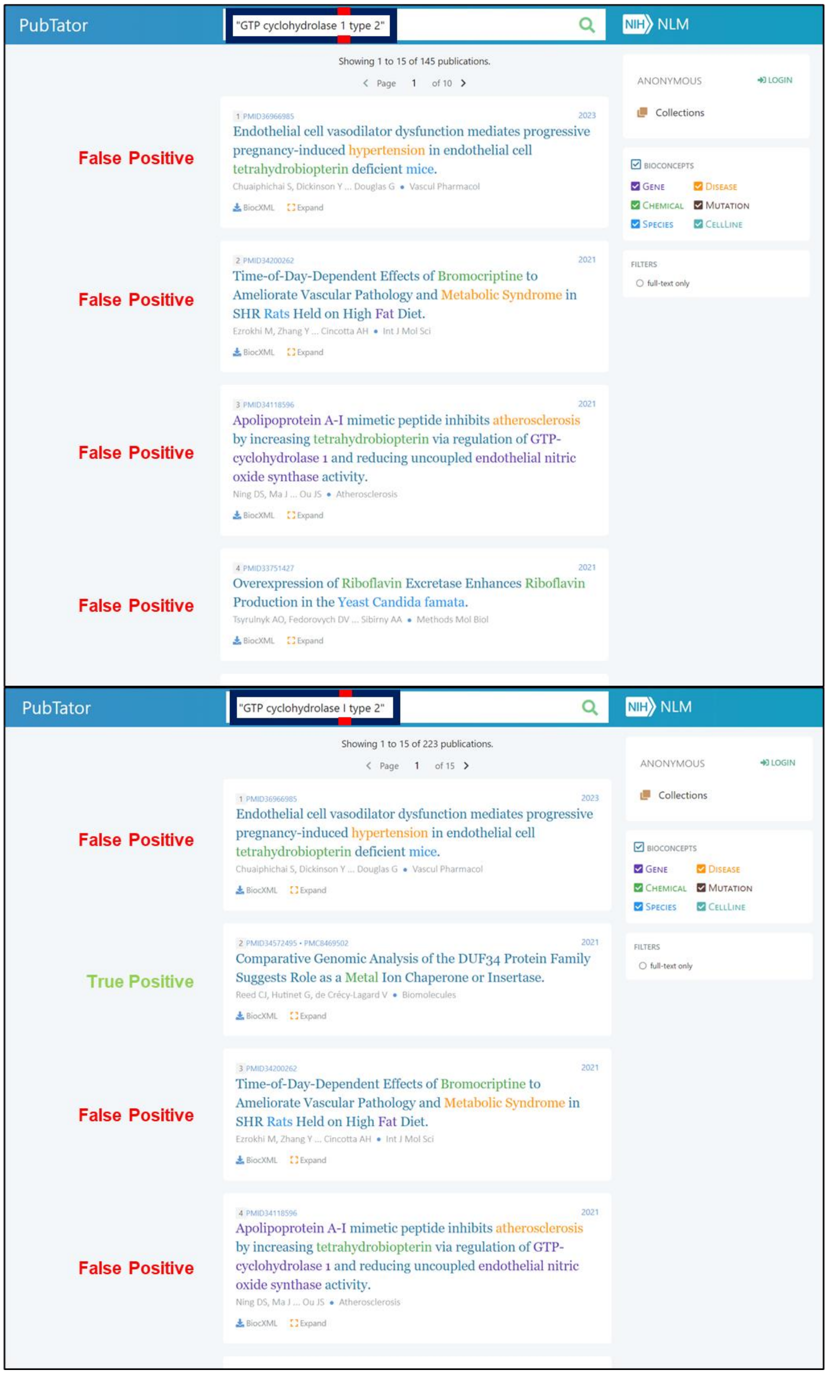

Figure S7. FlaGs (WebFlaGs) example outputs for NCBI BLASTp results for COG0327 (DUF34) family member of *E. coli*, YbgI (P0AFP6); list of RefSeq Accession IDs was used as the WebFlaGs input. a) Shows the PDF output showing the gene neighborhoods of the input sequences; cluster information is also available in text format accompanying this graphical output (not shown). b) Flanking genes tree graphical result option 1. c) Flanking genes tree graphical result option 2. d) MAFFT sequence alignment tree graphical result. The latter panels, b-d, are available as components constituting the exportable zipped folder available upon FlaGs output receipt.

a.

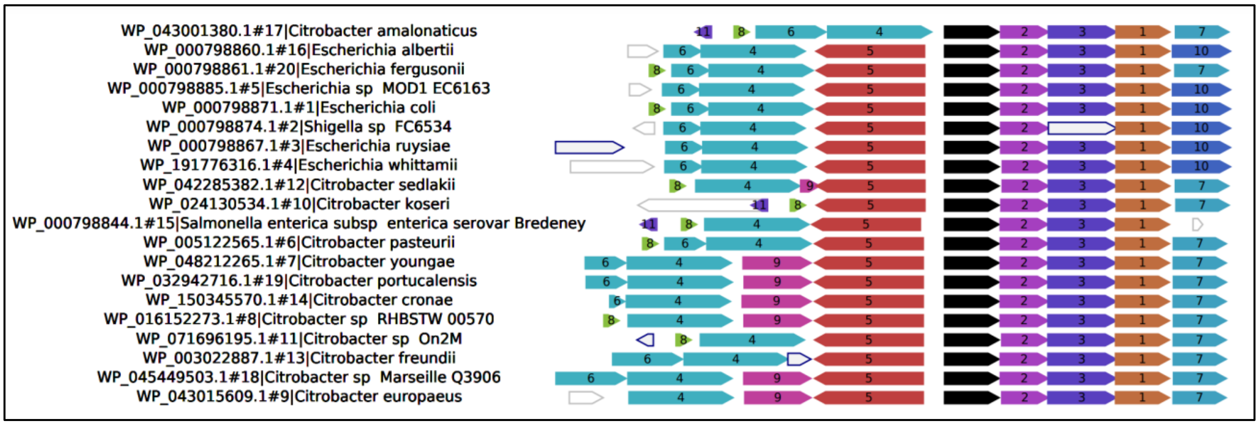

b.

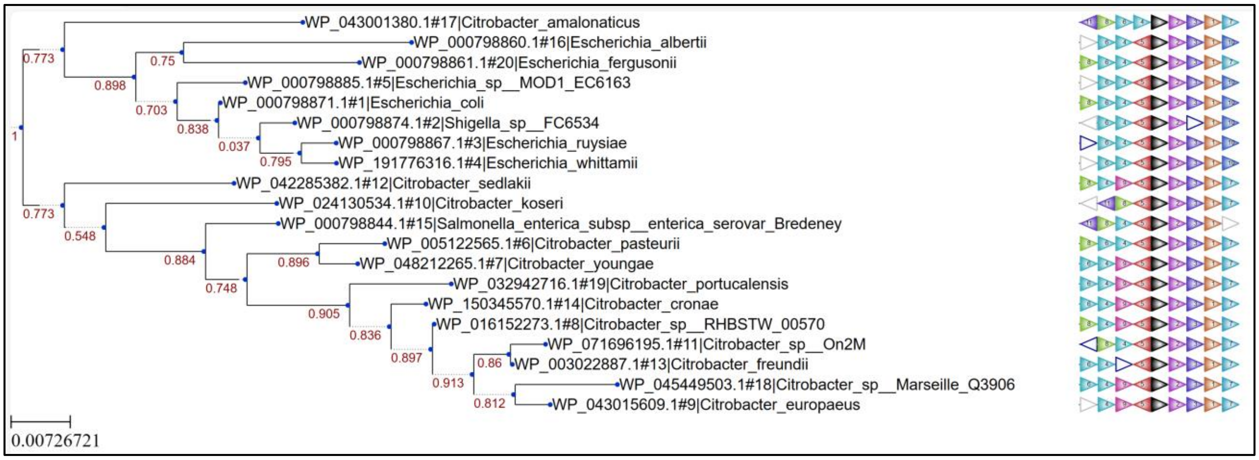

c.

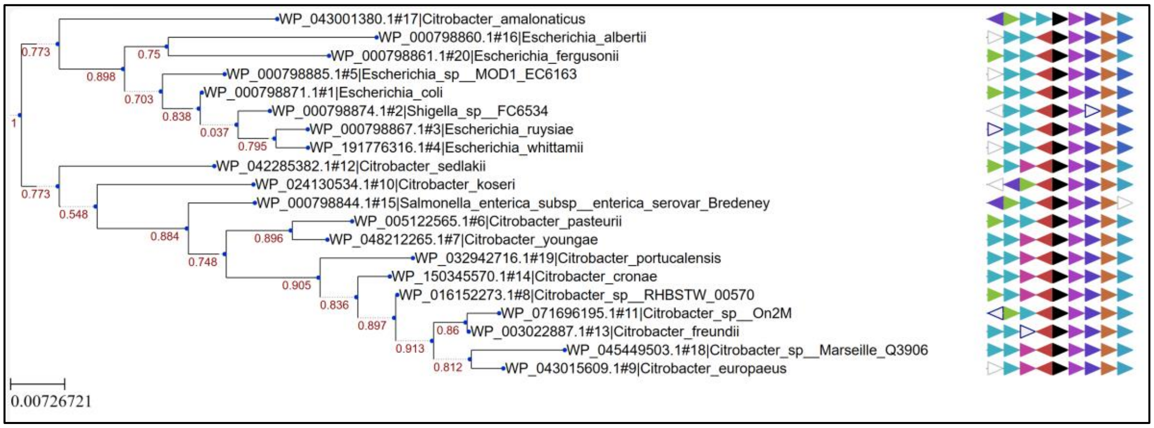
WP_043001380.1#17|Citrobacter_amalonaticus
WP_000798860.1#16|Escherichia_albertii
WP_000798861.1#20|Escherichia_fergusonii
WP_000798885.1#5|Escherichia_sp__MOD1_EC6163
WP_000798871.1#1|Escherichia_coli
WP_000798874.1#2|Shigella_sp__FC6534
WP_000798867.1#3|Escherichia_ruysiae
WP_191776316.1#4|Escherichia_whittamii
WP_042285382.1#12|Citrobacter_sedlakii
WP_024130534.1#10|Citrobacter_koseri
WP_000798844.1#15|Salmonella_enterica_subsp__enterica_serovar_Bredeney
WP_005122565.1#6|Citrobacter_pasteurii
WP_048212265.1#7|Citrobacter_youngae
WP_032942716.1#19|Citrobacter_portucalensis
WP_150345570.1#14|Citrobacter_cronae
WP_016152273.1#8|Citrobacter_sp__RHBSTW_00570
WP_071696195.1#11|Citrobacter_sp__On2M
WP_003022887.1#13|Citrobacter_freundii
WP_045449503.1#18|Citrobacter_sp__Marseille_Q3906
WP_043015609.1#9|Citrobacter_europaeus
0.00726721

d.

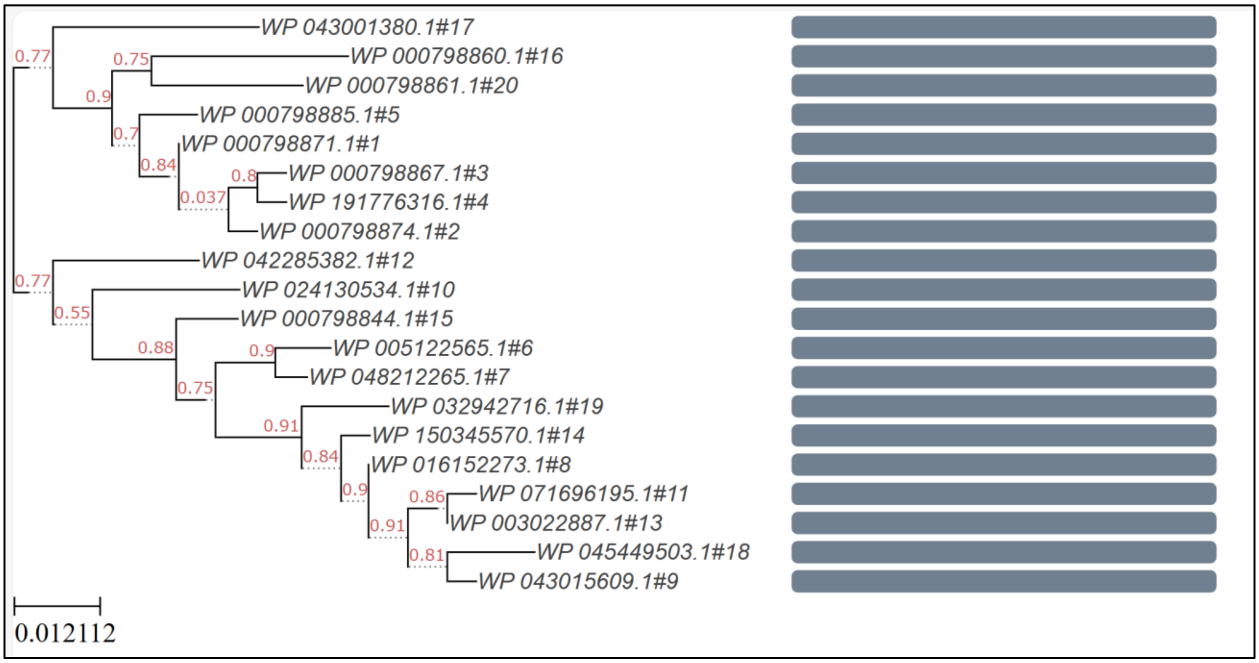
WP 043001380.1#17
WP 000798860.1#16
WP 000798861.1#20
WP 000798885.1#5
WP 000798871.1#1
WP 000798867.1#3
WP 191776316.1#4
WP 000798874.1#2
WP 042285382.1#12
WP 024130534.1#10
WP 000798844.1#15
WP 005122565.1#6
WP 048212265.1#7
WP 032942716.1#19
WP 150345570.1#14
WP 016152273.1#8
WP 071696195.1#11
WP 003022887.1#13
WP 045449503.1#18
WP 043015609.1#9
0.012112

Figure S8. EFI-GNT

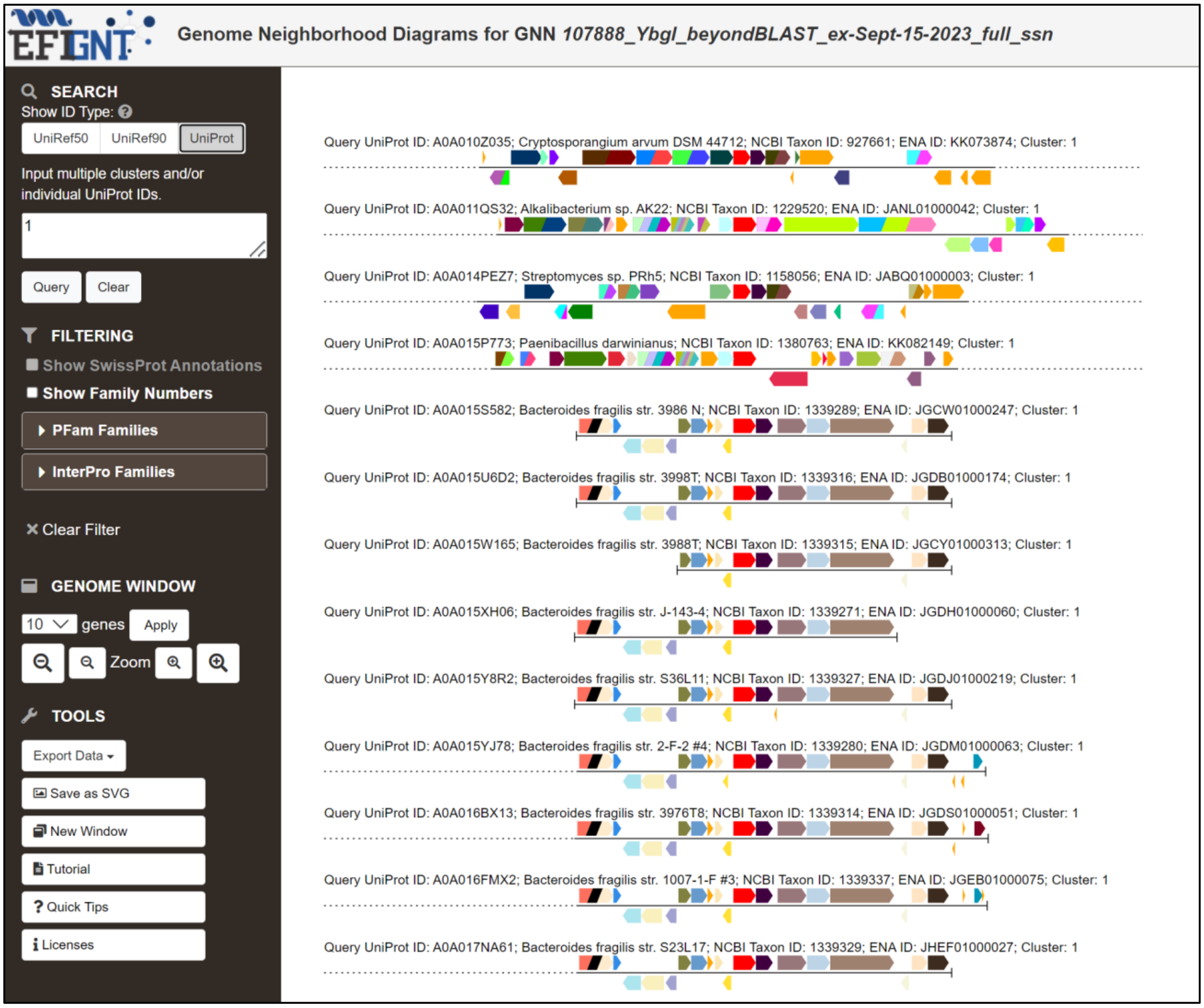

Figure S9. Annotree example: single family, PF02591, in bacteria.

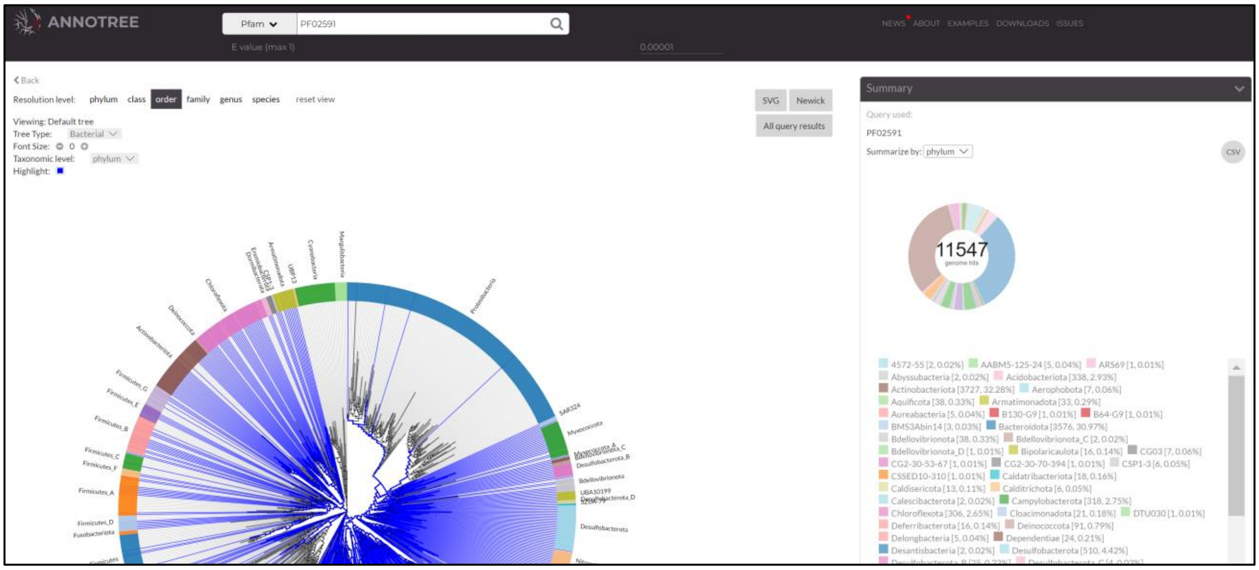

Figure S10. COGNAT COG-based gene neighborhood and physical clustering tool example output for COG0327 (DUF34 protein family). a) shows the raw output, while b) shows a portion of the pdf export made available via link-out download.

a.

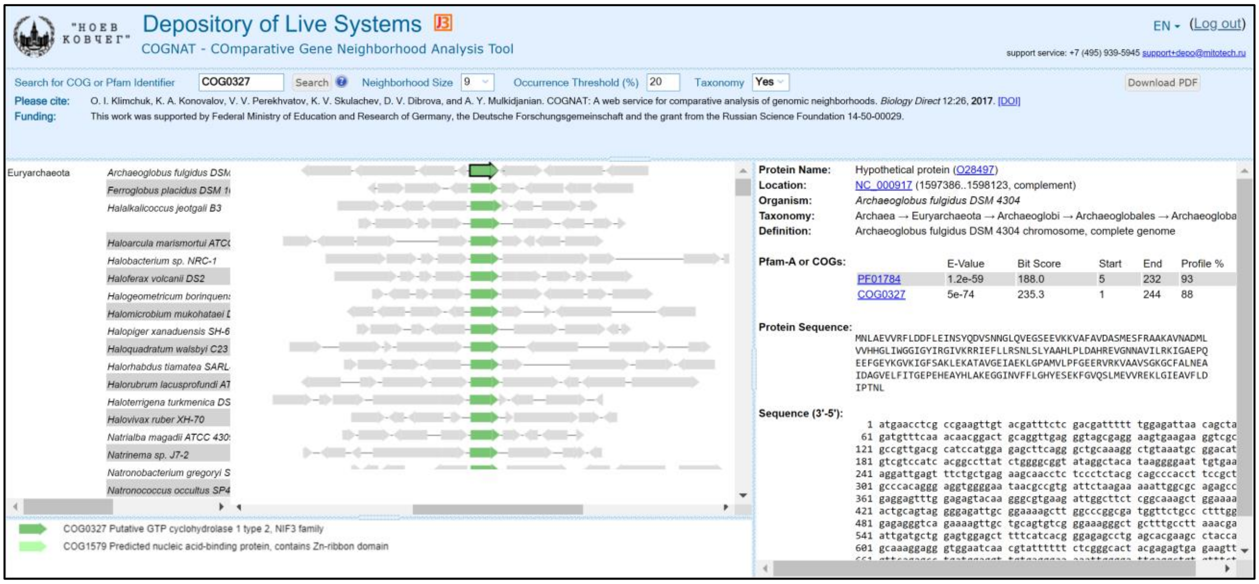

b.

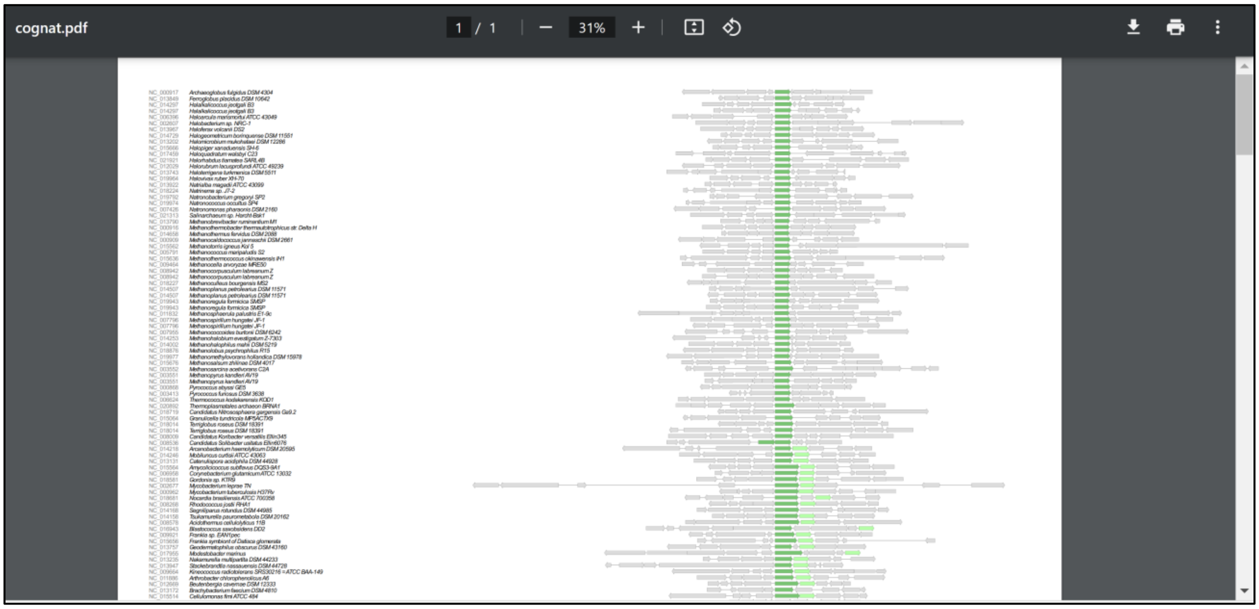

Figure S11. SubtiWiki (CoreWiki) beta feature of the database's Genomic Neighborhood Comparison viewer of the DUF34 homolog of B. subtilis, YqfO (BSU_25170).

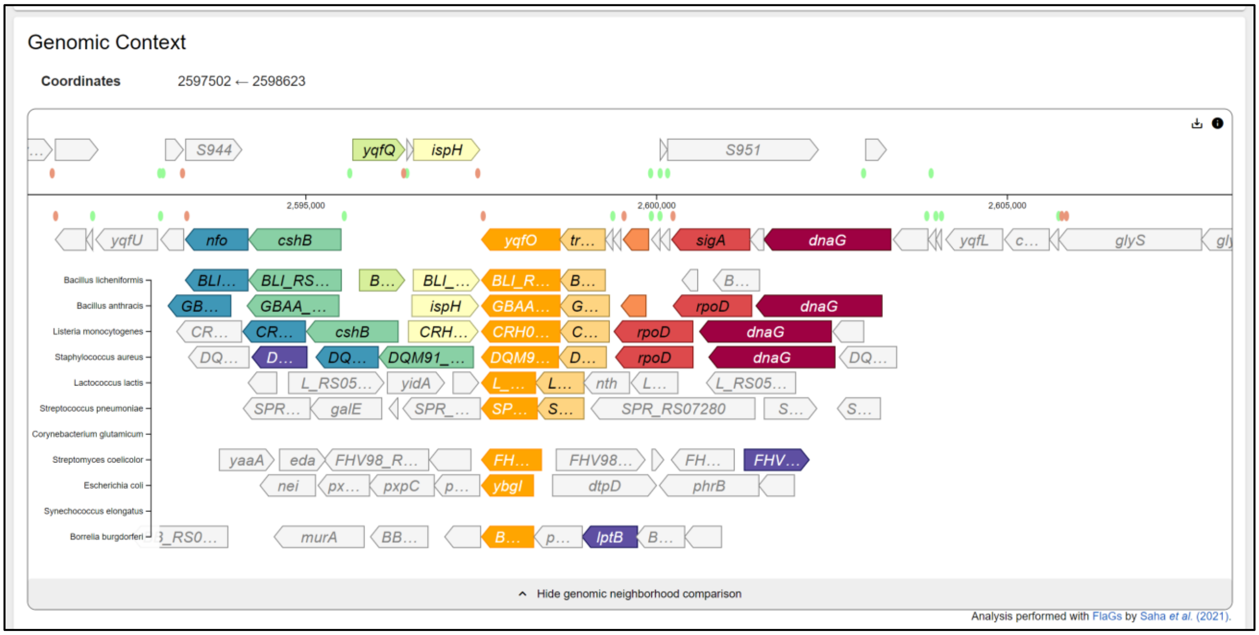

Figure S12. Example tree output of MicrobesOnline.

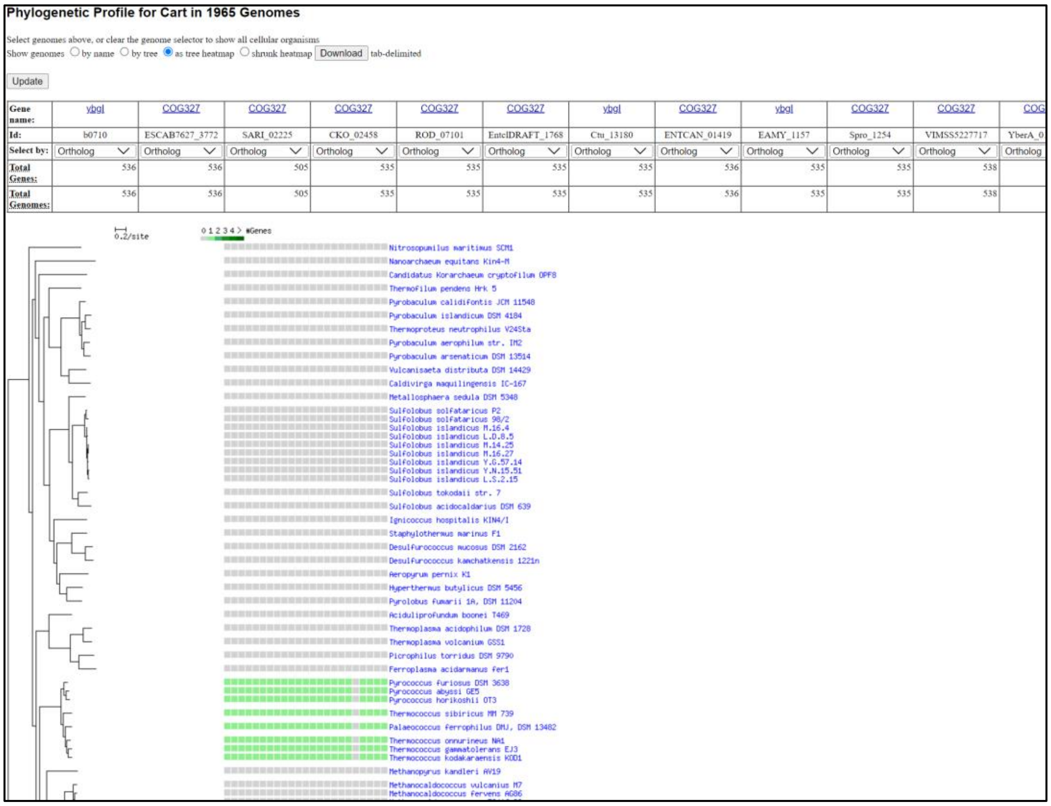

Figure S13. Screengrab demonstrating different "filters" for target family recognition, particularly used differentially with multiple targets (MicrobesOnline).

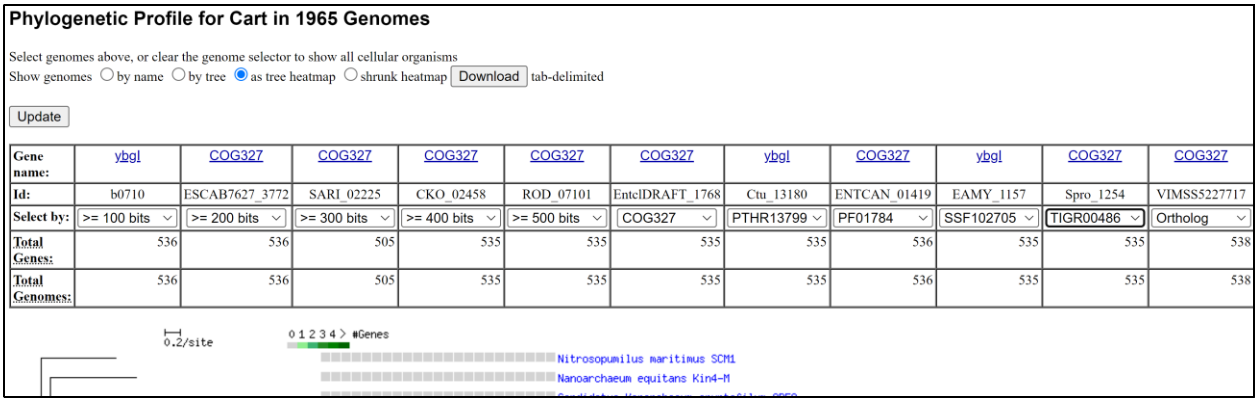

Gene name:	ybgI	COG327	COG327	COG327	COG327	COG327	ybgI	COG327	ybgI	COG327	COG327
Id:	b0710	ESCAB7627_3772	SARI_02225	CKO_02458	ROD_07101	EntclDRAFT_1768	Ctu_13180	ENTCAN_01419	EAMY_1157	Spro_1254	VIMSS5227717
Select by:	>= 100 bits	>= 200 bits	>= 300 bits	>= 400 bits	>= 500 bits	COG327	PTHR13799	PF01784	SSF102705	TIGR00486	Ortholog
Total Genes:	536	536	505	535	535	535	535	536	535	535	538
Total Genomes:	536	536	505	535	535	535	535	536	535	535	538

Figure S14. Screengrab demonstrating navigation to family-specific trees in MicrobesOnline via the "Gene Info" tab of each entry.

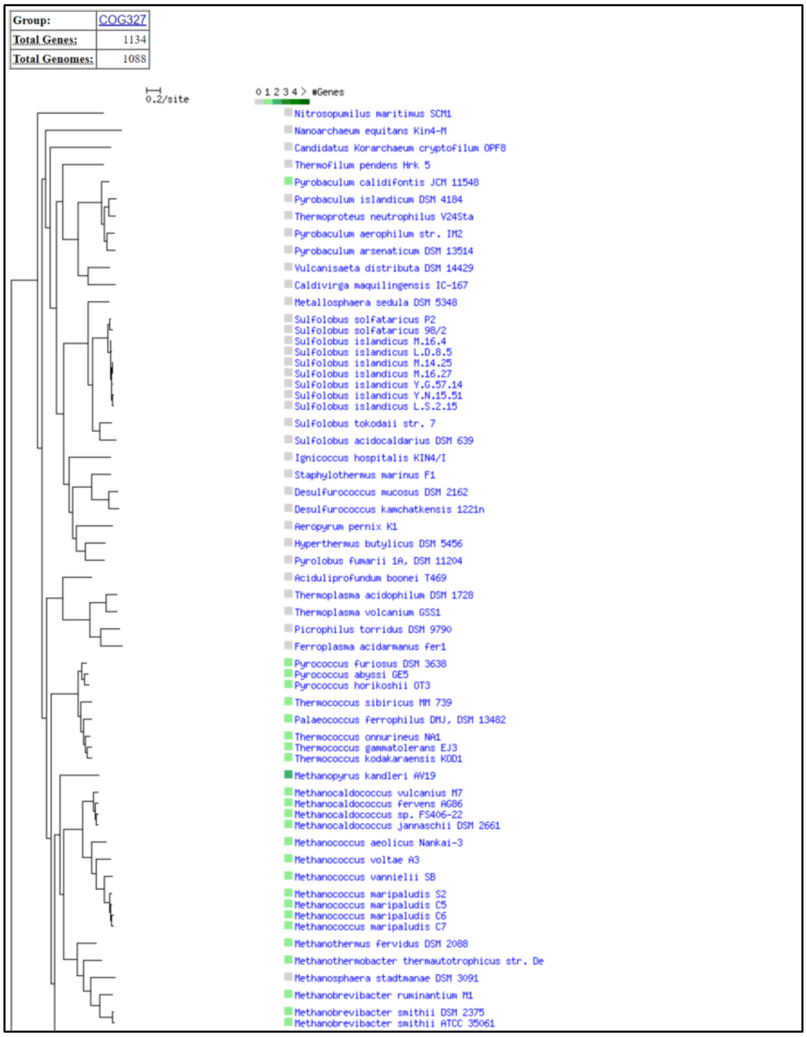

Group:	COG327
Total Genes:	1134
Total Genomes:	1088

Figure S15. “Families” (COGs) in STRING search tool options.

a. Input, multiple protein names (release, 12.0)

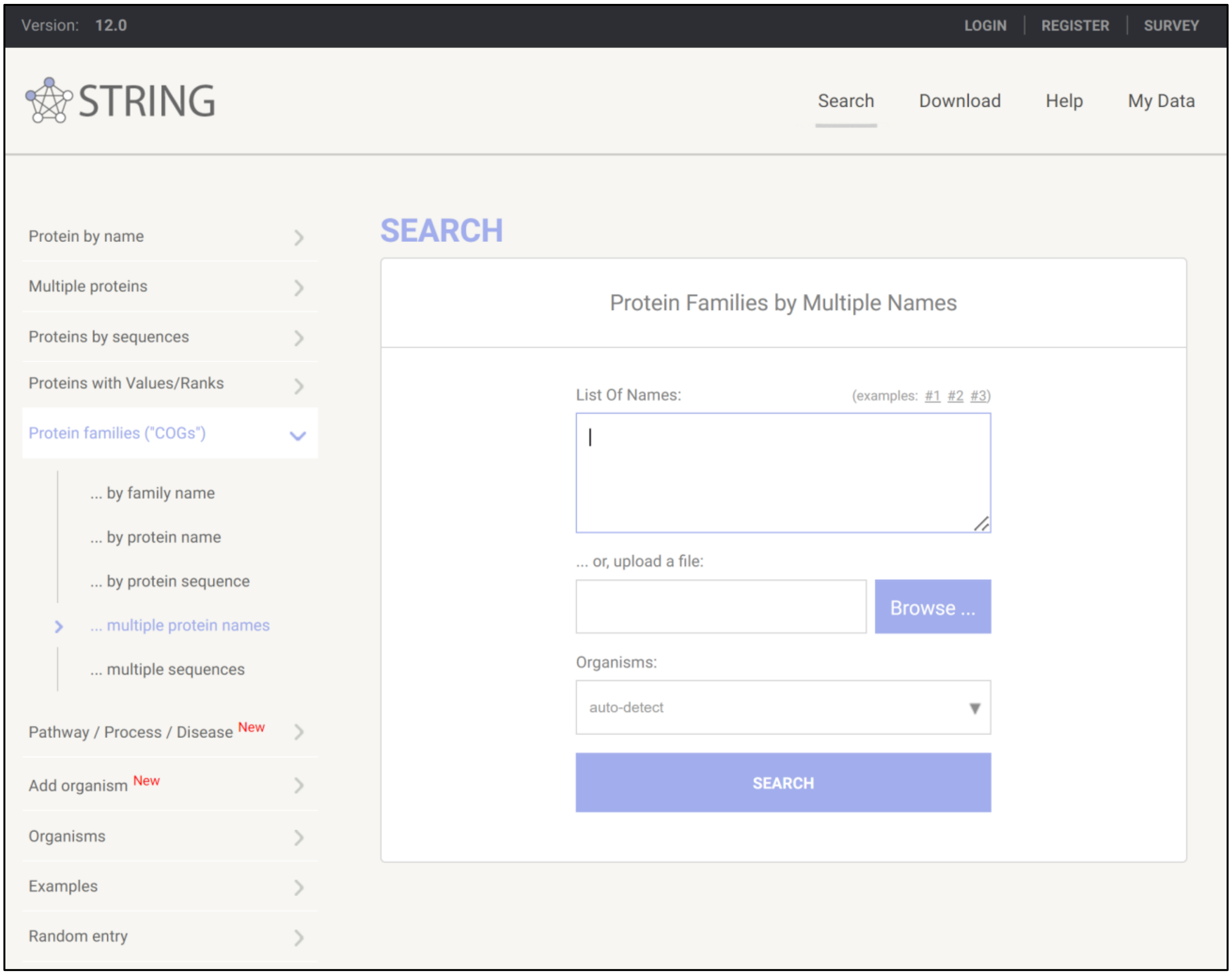

b. STRING network output for the physically-interacting proteins DUF34 family protein P9WFM1 (COG0327) and P9WLH3 (COG1579) of *Mycobacterium tuberculosis* (strain ATCC 25618 / H37Rv) via "multiple family names" type query; high-confidence (0.700) minimum required interaction score; no more than 50 interactors, max number of interactors to show for first shell; no more than 5 interactors, max number of interactions to show for second shell.

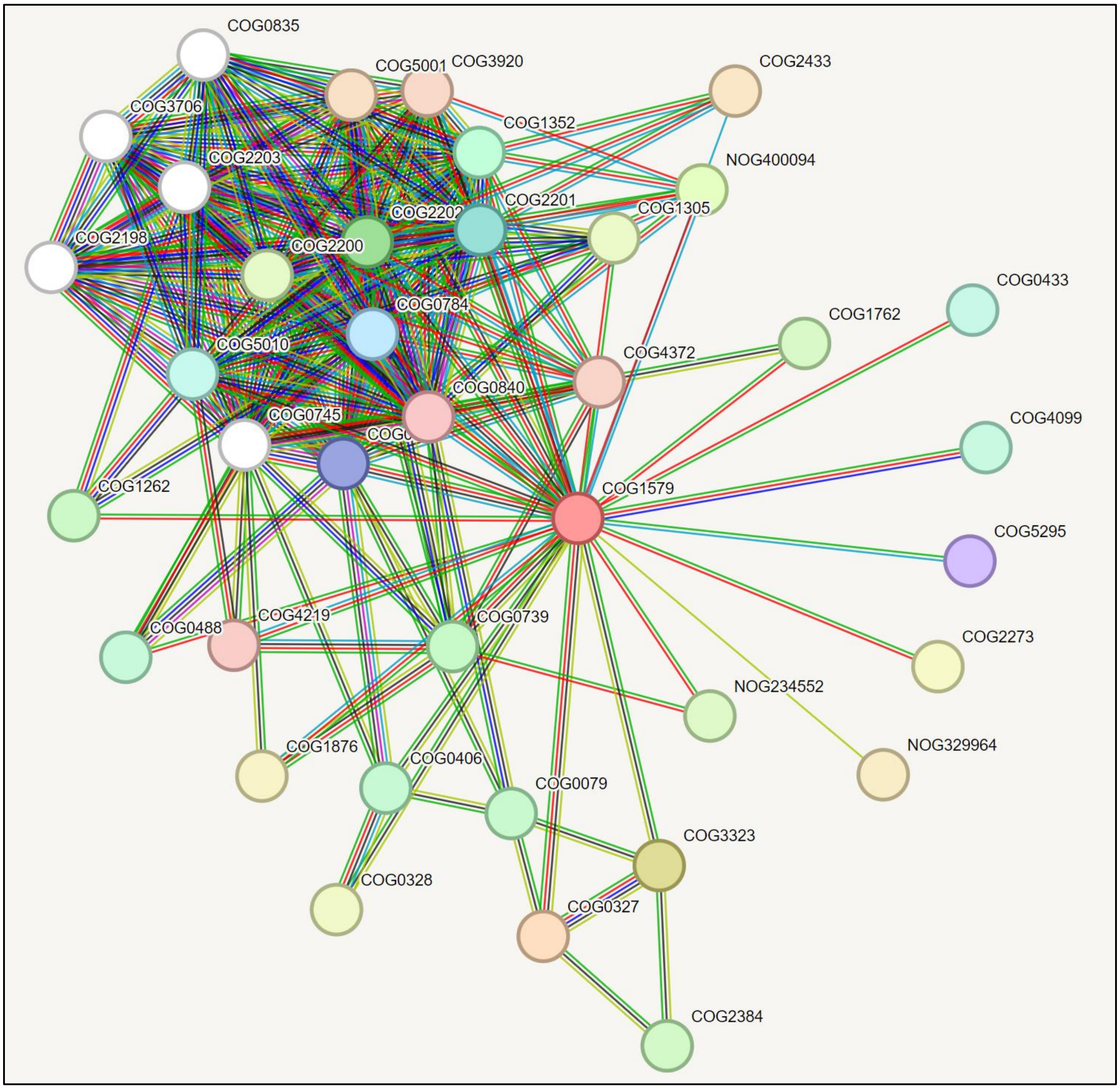

c. STRING output for the co-occurrence view for COG0327 (DUF34 family homolog, P9WFM1) and COG2072 (P9WLH3) (phylogeny collapses/expansions that are shown were generated by default).

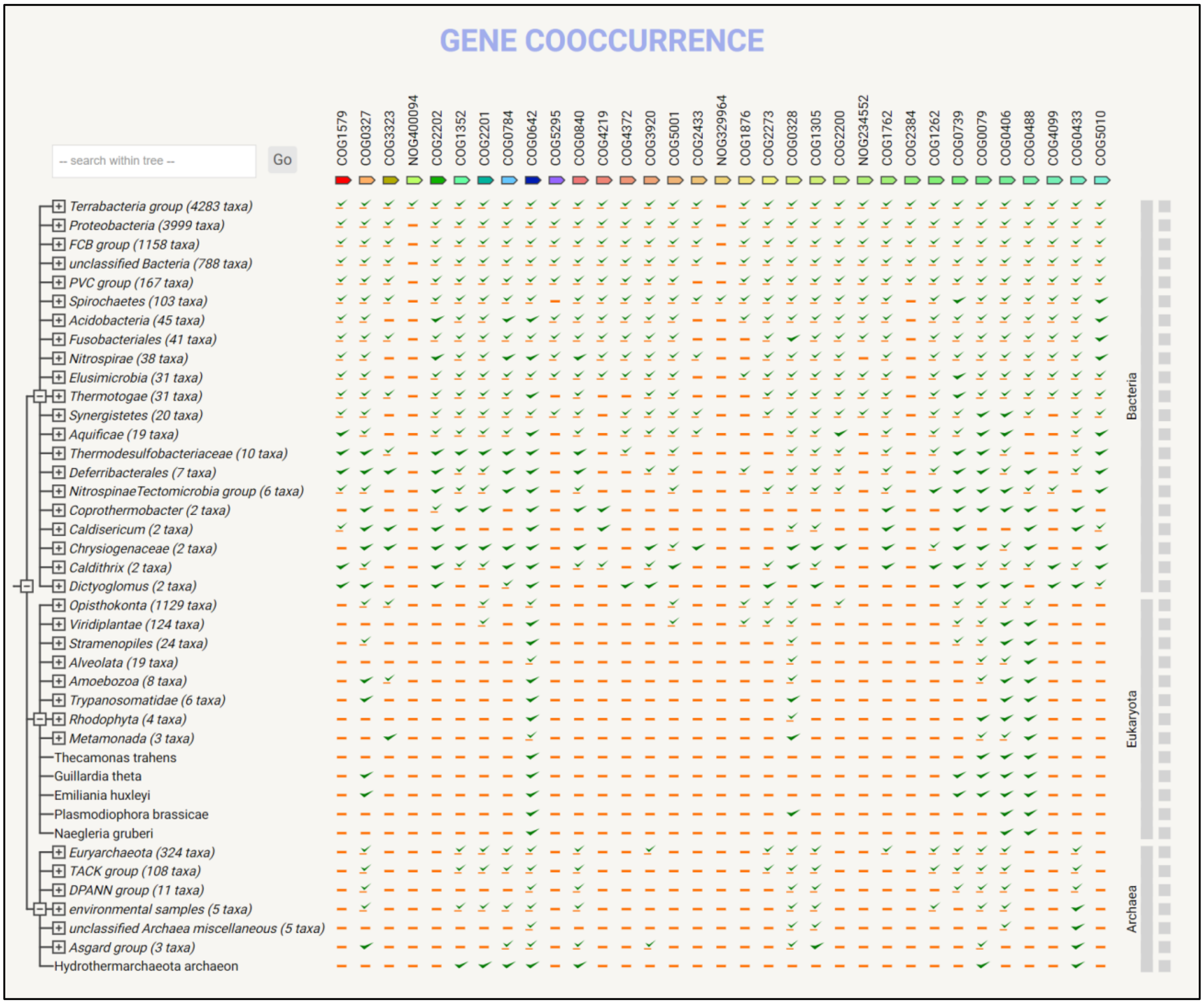

Figure S16. KO identifiers being supplied to KEGG orthology tool (multiple families, limited genome customization).

a. Output for TsaE (K06925, ubiquitous protein family) and COG1579 (K07164) in KEGG Orthology K number search.

Ortholog table

Grp	Genus	Organism	K06925 (tsaE)[6863]	K07164 (K07164)[2322]
B.GamE	Escherichia	eco	b4168	
B.GamE	Escherichia	ecj	JW4126	
B.GamE	Escherichia	ecd	ECDH10B_4363	
B.GamE	Escherichia	ebw	BWG_3880	
B.GamE	Escherichia	ecok	ECMDS42_3610	
B.GamE	Escherichia	ece	Z5775	
B.GamE	Escherichia	ecs	ECs_5144	
B.GamE	Escherichia	ecf	ECH74115_5684	
B.GamE	Escherichia	etw	ECSP_5268	
B.GamE	Escherichia	elx	CDCO157_4830	
B.GamE	Escherichia	eoi	ECO111_5046	
B.GamE	Escherichia	eoj	ECO26_5334	
B.GamE	Escherichia	eoh	ECO103_4961	
B.GamE	Escherichia	ecoo	ECRM13514_5432	
B.GamE	Escherichia	ecoh	ECRM13516_5179	
B.GamE	Escherichia	esl	O3K_22805	
B.GamE	Escherichia	eso	O3O_02580	
B.GamE	Escherichia	esm	O3M_22710	
B.GamE	Escherichia	eck	EC55989_4723	
B.GamE	Escherichia	ecg	E2348C_4491	
B.GamE	Escherichia	eok	G2583_4995	
B.GamE	Escherichia	elr	ECO55CA74_23945	
B.GamE	Escherichia	elh	ETEC_4514	
B.GamE	Escherichia	ecw	EcE24377A_4725	
B.GamE	Escherichia	eun	UMNK88_5106	
B.GamE	Escherichia	ecp	ECP_4413	
B.GamE	Escherichia	ena	ECNA114_4384	
B.GamE	Escherichia	ecos	EC958_4655	
B.GamE	Escherichia	ecv	APECO1_2223	
B.GamE	Escherichia	ecoa	APECO78_01770	
B.GamE	Escherichia	ecx	EcHS_A4410	
B.GamE	Escherichia	ecm	EcSMS35_4639	
B.GamE	Escherichia	ecy	ECSE_4465	
B.GamE	Escherichia	ecr	ECIAI1_4401	
B.GamE	Escherichia	ecq	ECED1_4953	
B.GamE	Escherichia	eum	ECUMN_4701	
B.GamE	Escherichia	ect	ECIAI39_4632	
B.GamE	Escherichia	eoc	CE10_4907	
B.GamE	Escherichia	ebr	ECB_04035	
B.GamE	Escherichia	ebl	ECD_04035	
B.GamE	Escherichia	ebe	B21_03997	
B.GamE	Escherichia	ebd	ECBD_3866	
B.GamE	Escherichia	eci	UTI89_C4768	
B.GamE	Escherichia	eih	ECOK1_4682	
B.GamE	Escherichia	ecz	ECS88_4754	
B.GamE	Escherichia	ecc	c5252	
B.GamE	Escherichia	elo	EC042_4641	
B.GamE	Escherichia	eln	NRG857_21185	
B.GamE	Escherichia	ese	ECSF_4054	
B.GamE	Escherichia	ecl	EcolC_3845	
B.GamE	Escherichia	eko	EKO11_4144	
B.GamE	Escherichia	ekf	KO11_22465	
B.GamE	Escherichia	eab	ECABU_c47260	
B.GamE	Escherichia	edh	EcDH1_3825	
B.GamE	Escherichia	edj	ECDH1ME8569_402	
B.GamE	Escherichia	elu	UM146_21075	
B.GamE	Escherichia	elw	ECW_m4530	
[illegible]	[illegible]	[illegible]	[illegible]	

b. Input for KEGG Orthology K number search (ortholog table).

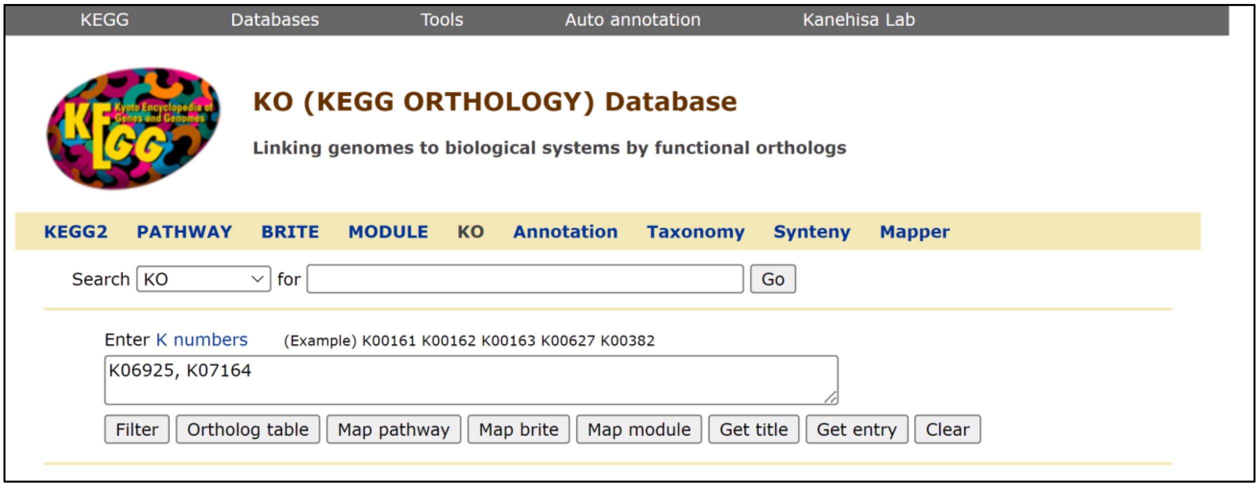

Figure S17. Example CAGECAT output using DUF34 and COG1579 homolog sequences: MSMEG_4307 (NCBI-ProteinID: ABK73705); MSMEG_4306 (NCBI-ProteinID: ABK70599). This figure was generated after using cblaster then clinker applications as part of a tandem suite of tools, namely the "compared to query" visualization.

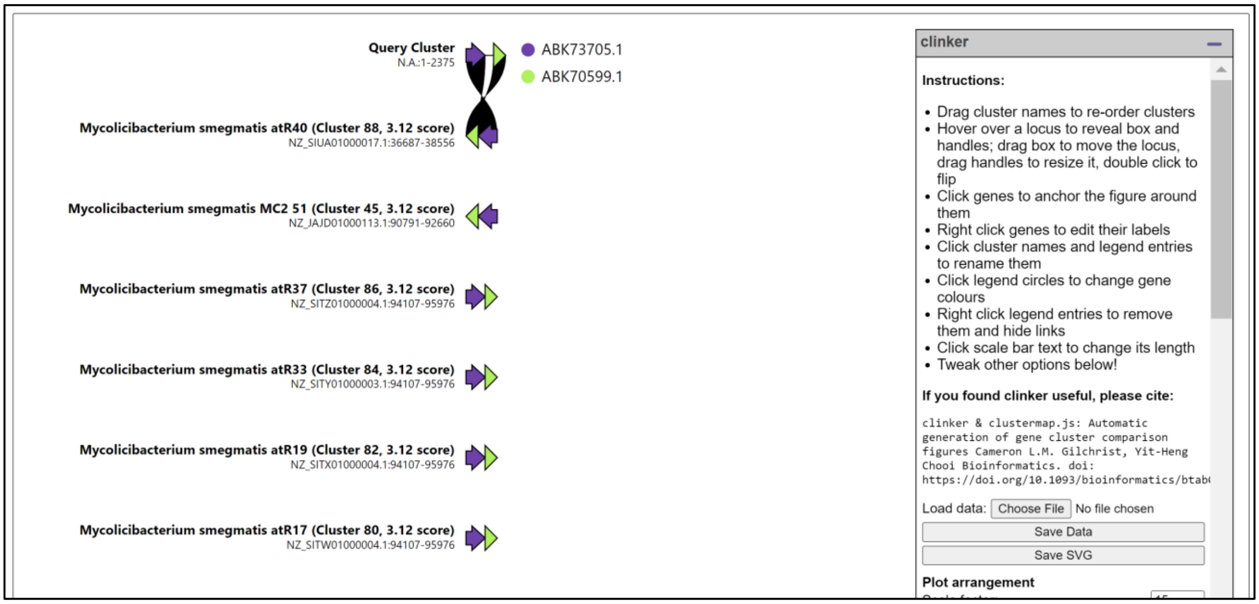

Figure S18. Example output for FunCoup using "YbgI" of *E. coli*. While other interaction/correlational data is present, operons are included in each subset.

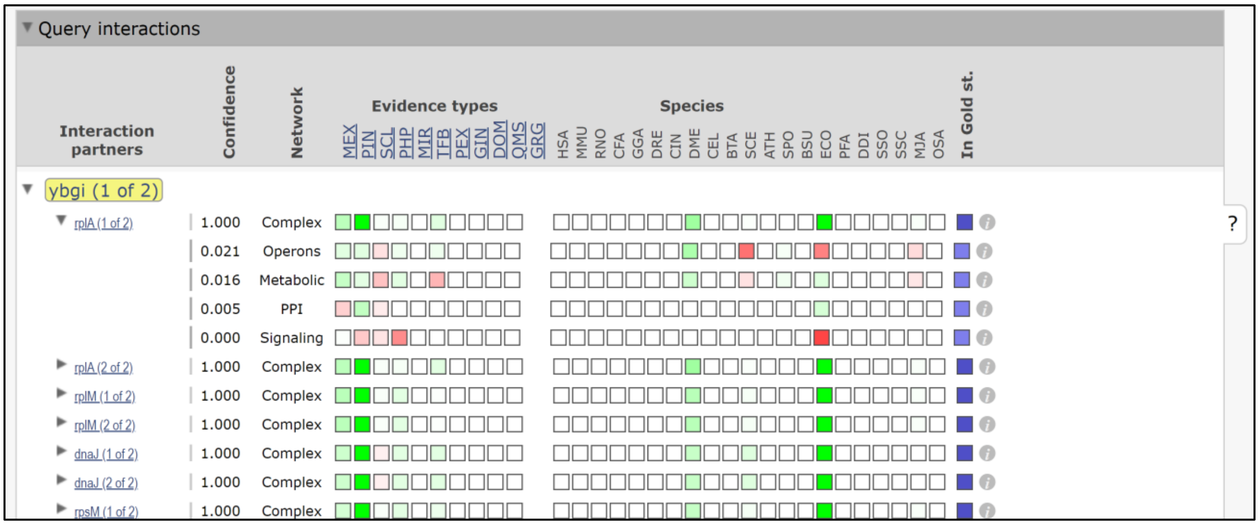

Figure S19. BV-BRC protein family sorter, general output example (a) with additional viewer examples: b) subsystems; c) pathways – KEGG Map; d) pathways – Heatmap; e) families.

a. Initial Output Files (all)

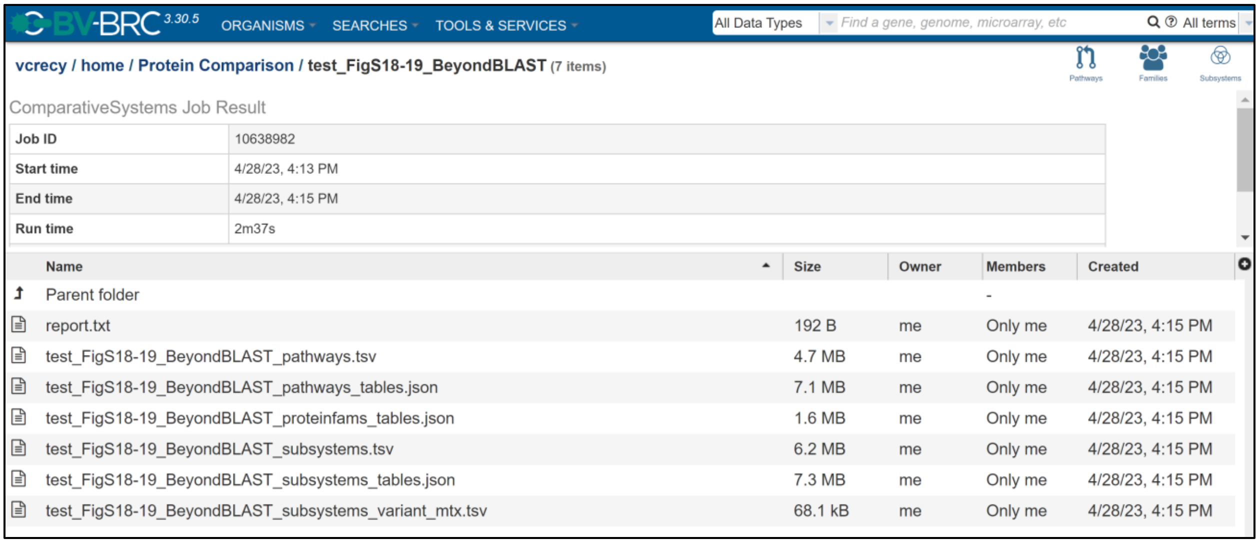

b. Subsystems (overview). Subsystems (via the “Subsystems” tab adjacent to the “Subsystems Overview” tab) can then be selected individually or together (>1) for either mapping for the former case or grouping for the latter.

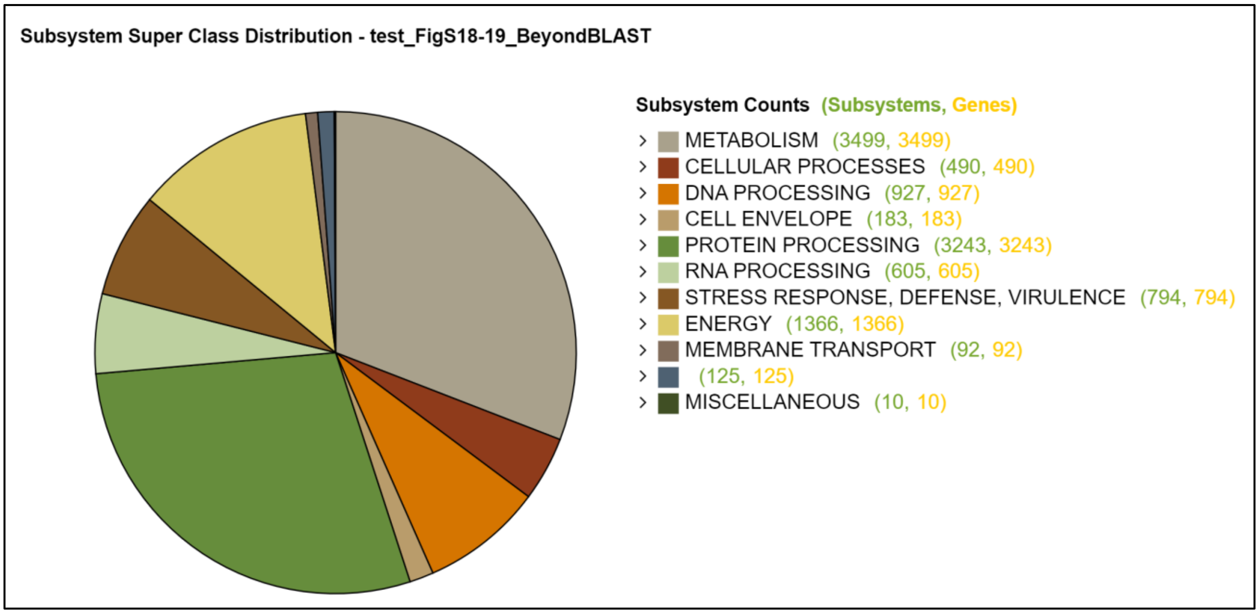

c. Pathways – KEGG Map

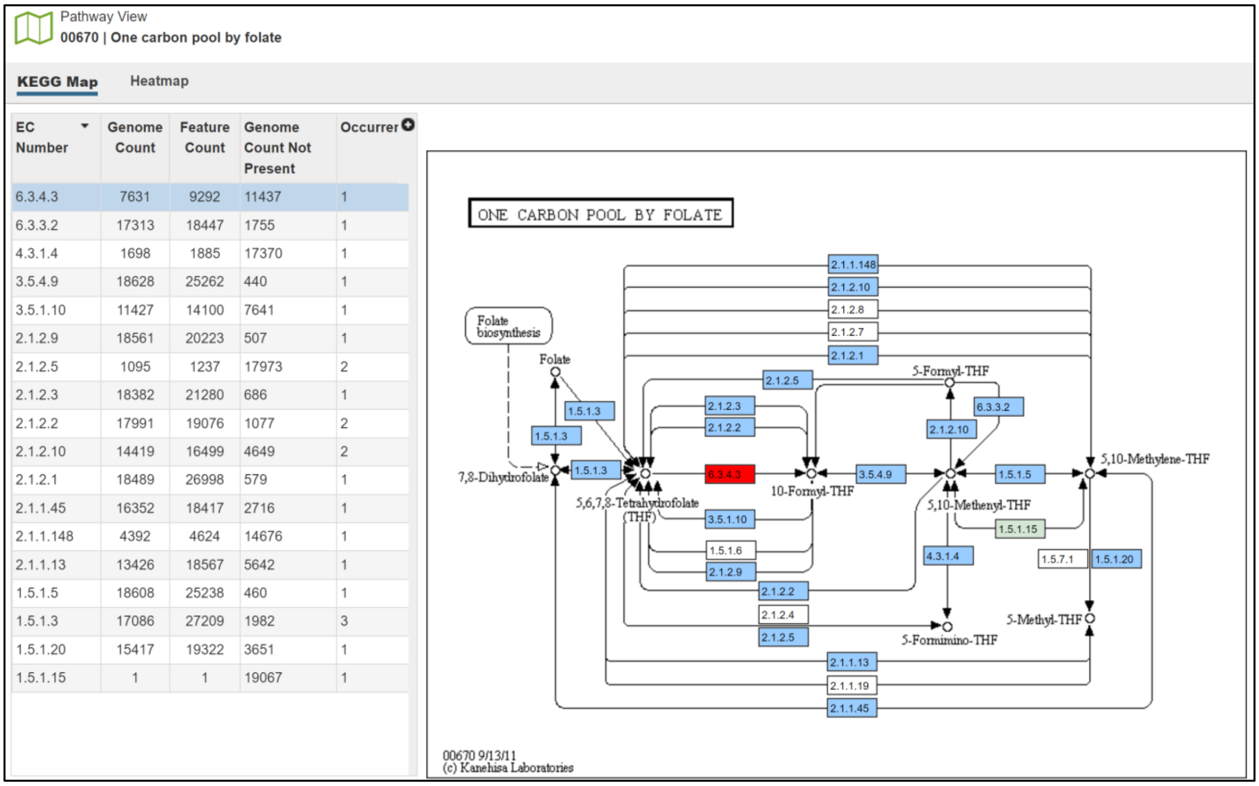

EC Number	Genome Count	Feature Count	Genome Count Not Present	Occurrer
6.3.4.3	7631	9292	11437	1
6.3.3.2	17313	18447	1755	1
4.3.1.4	1698	1885	17370	1
3.5.4.9	18628	25262	440	1
3.5.1.10	11427	14100	7641	1
2.1.2.9	18561	20223	507	1
2.1.2.5	1095	1237	17973	2
2.1.2.3	18382	21280	686	1
2.1.2.2	17991	19076	1077	2
2.1.2.10	14419	16499	4649	2
2.1.2.1	18489	26998	579	1
2.1.1.45	16352	18417	2716	1
2.1.1.148	4392	4624	14676	1
2.1.1.13	13426	18567	5642	1
1.5.1.5	18608	25238	460	1
1.5.1.3	17086	27209	1982	3
1.5.1.20	15417	19322	3651	1
1.5.1.15	1	1	19067	1

d. Pathways – Heatmap

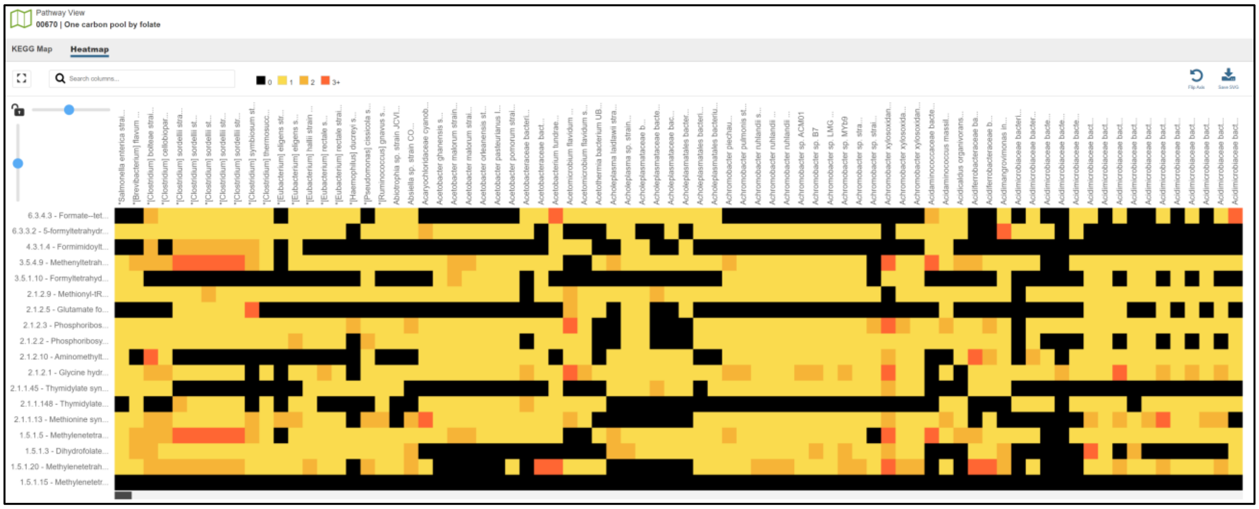

e. Families (output)

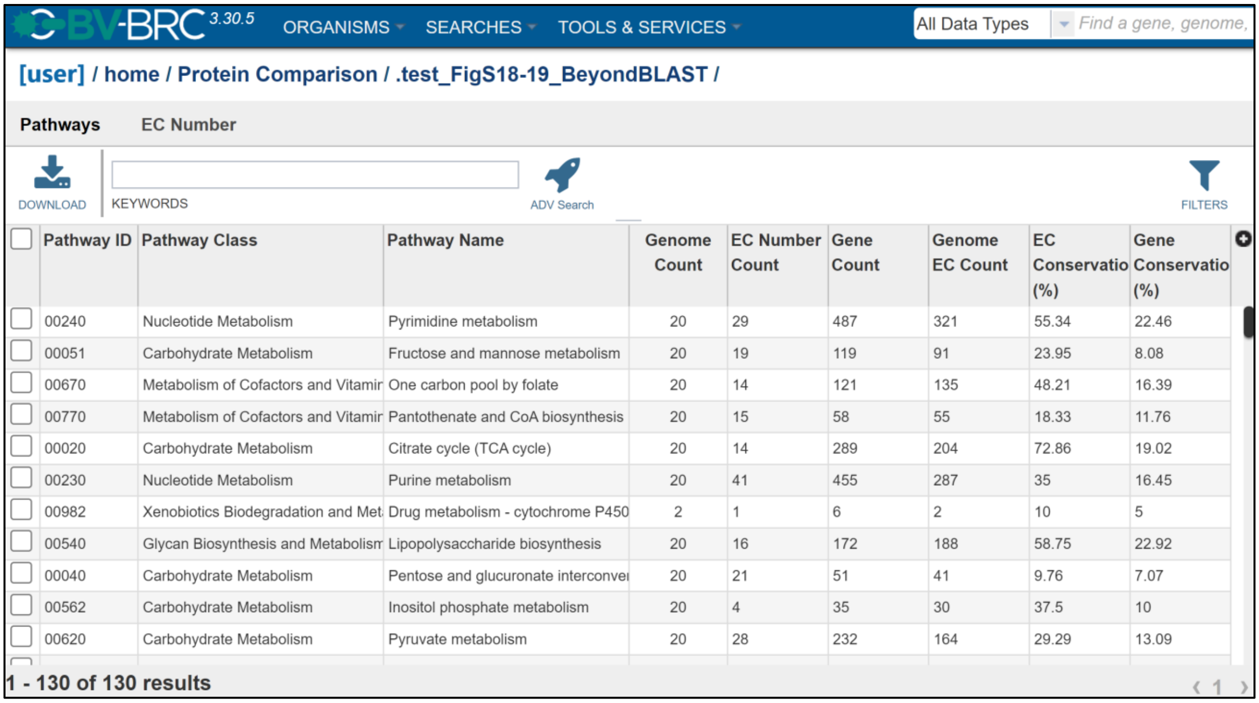

	Pathway ID	Pathway Class	Pathway Name	Genome Count	EC Number Count	Gene Count	Genome EC Count	EC Conservatio (%)	Gene Conservatio (%)
	00240	Nucleotide Metabolism	Pyrimidine metabolism	20	29	487	321	55.34	22.46
	00051	Carbohydrate Metabolism	Fructose and mannose metabolism	20	19	119	91	23.95	8.08
	00670	Metabolism of Cofactors and Vitamir	One carbon pool by folate	20	14	121	135	48.21	16.39
	00770	Metabolism of Cofactors and Vitamir	Pantothenate and CoA biosynthesis	20	15	58	55	18.33	11.76
	00020	Carbohydrate Metabolism	Citrate cycle (TCA cycle)	20	14	289	204	72.86	19.02
	00230	Nucleotide Metabolism	Purine metabolism	20	41	455	287	35	16.45
	00982	Xenobiotics Biodegradation and Met:	Drug metabolism - cytochrome P450	2	1	6	2	10	5
	00540	Glycan Biosynthesis and Metabolism	Lipopolysaccharide biosynthesis	20	16	172	188	58.75	22.92
	00040	Carbohydrate Metabolism	Pentose and glucuronate interconvei	20	21	51	41	9.76	7.07
	00562	Carbohydrate Metabolism	Inositol phosphate metabolism	20	4	35	30	37.5	10
	00620	Carbohydrate Metabolism	Pyruvate metabolism	20	28	232	164	29.29	13.09

1 - 130 of 130 results

Figure S20. BV-BRC protein family sorter output, Families.

a. Heatmap view of Families output. Green boxes highlight the text entry box and the heatmap tab selection. Text box provides search ability that is dependent upon annotation quality in database.

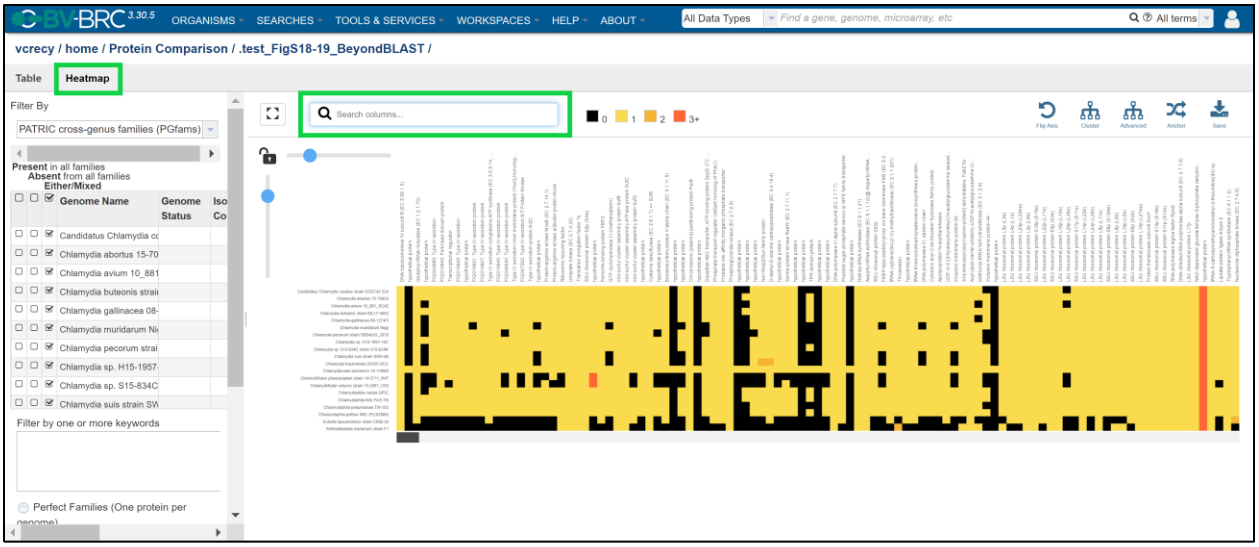

b. Table view of Families output. Purple boxes highlight the multiple-select checkboxes that a user may select for viewing in the accompanying heatmap or for export/grouping.

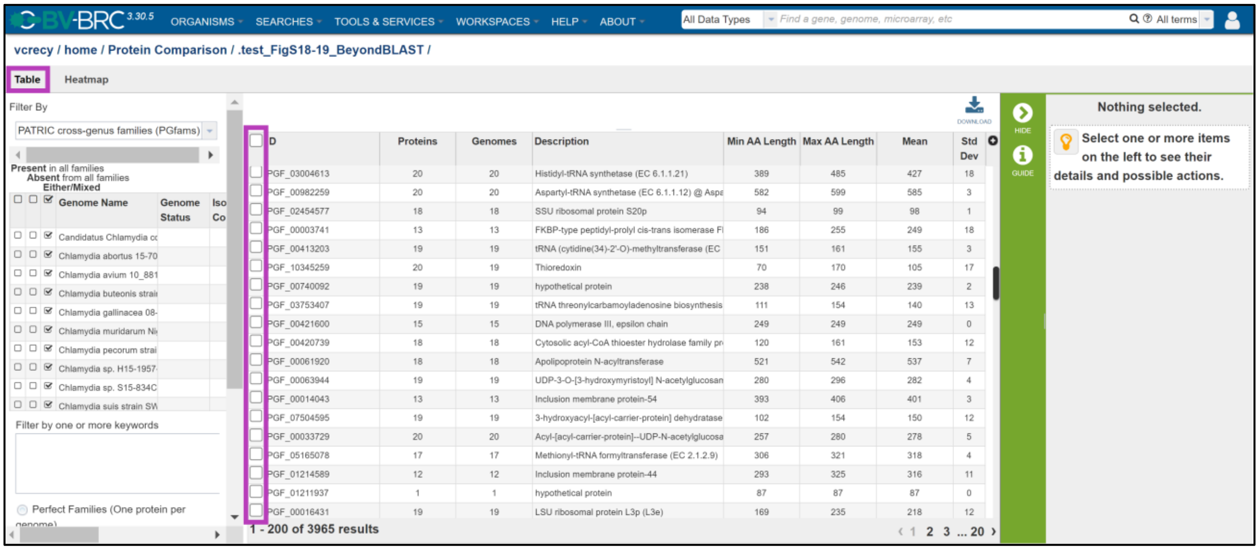

Figure S21. EggNOG Phylogenetic Profile tool example output using the site-provided example input data. At the time of writing, the tool seemed to be experiencing loading and/or query processing errors, failing to generate the intended product.

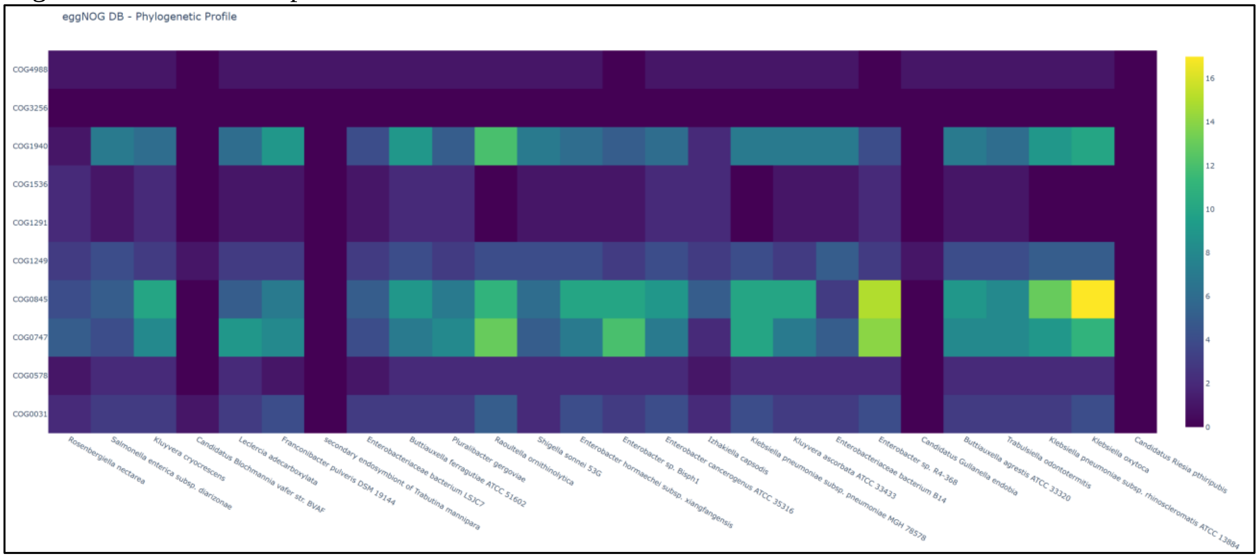

.

Figure S22. KBase workflow for generating a "gene tree" (as regarded by KBase) from several select genomes. Figure was directly adapted from a KBase 2020 phylogenomics online workshop diagram. Circles indicate data for input/outputs. Squares indicate applications within the KBase *"narrative"* (i.e., "narratives" in KBase are curated workflows of applications, as well as respective inputs and outputs [data objects], that can be created by users or, alternatively, made available to users by developers for implementation as example templates for common workflows).

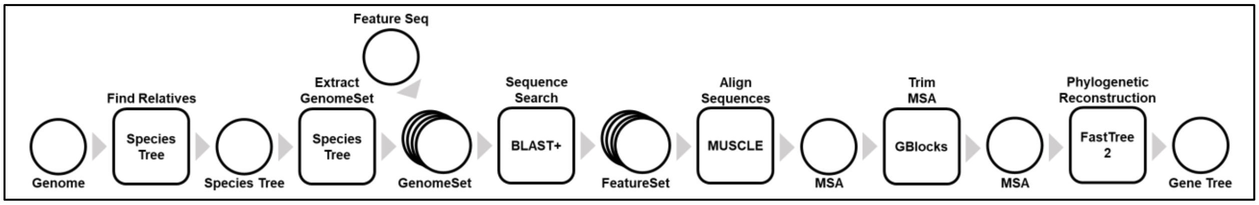

Figure S23. Genomic Context Visualizer (GeCoViz)

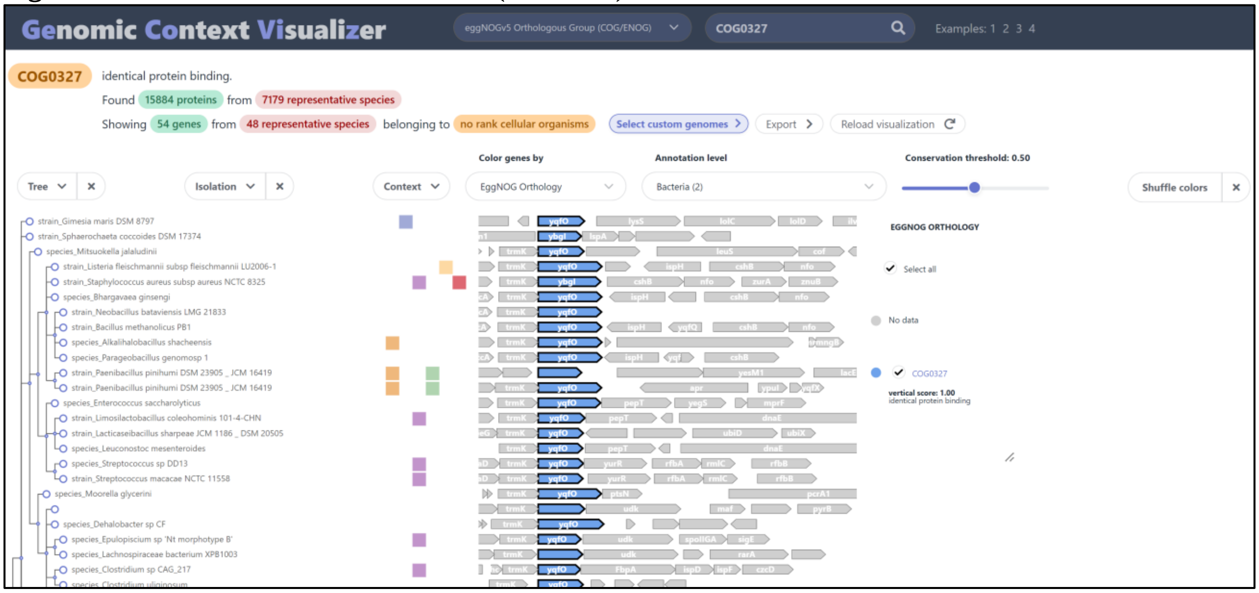

Figure S24. GizmoGene example output for the DUF34 homolog of *Mycobacterium tuberculosis* H37Rv_CG (BV-BRC feature ID: fig|1773.25616.peg.2443) using a custom set of BV-BRC genomes (62 Representative genomes, bacteria).

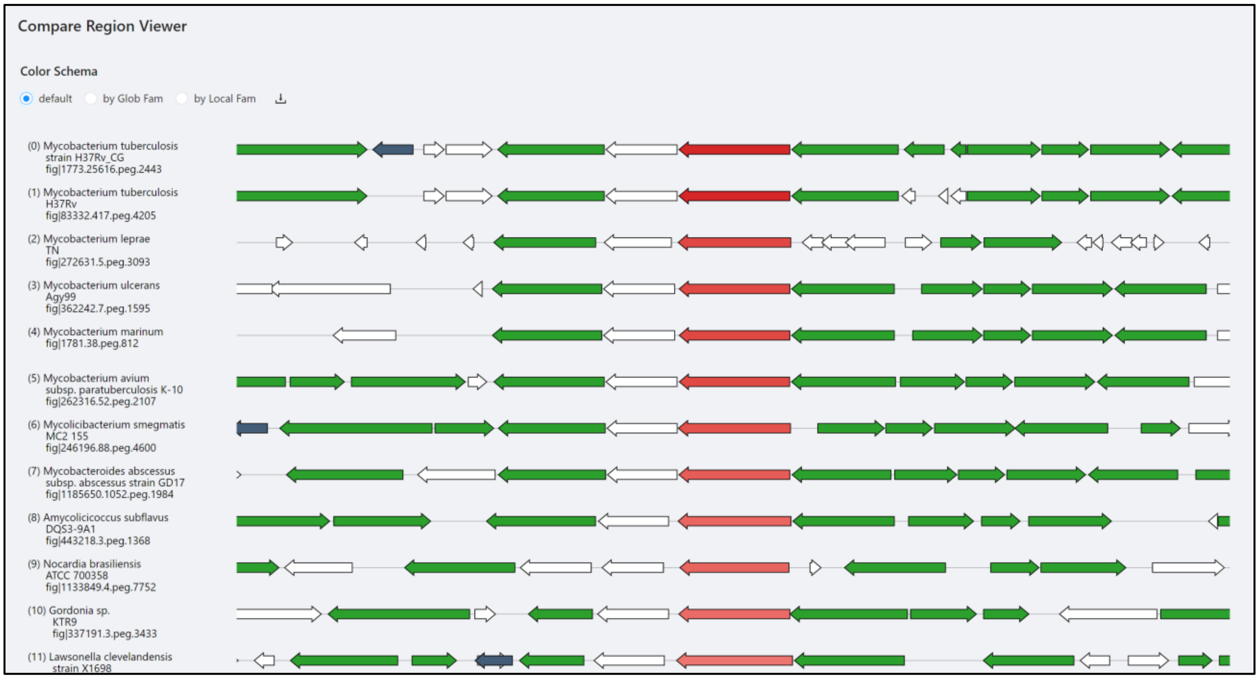

Supplementary References

1. Gat, O.; Lapidot, A.; Alchanati, I.; Regueros, C.; Shoham, Y. Cloning and DNA sequence of the gene coding for Bacillus stearothermophilus T-6 xylanase. *Appl. Environ. Microbiol.* **1994**, *60*, 1889–1896, doi:10.1128/AEM.60.6.1889-1896.1994.

2. Shulami, S.; Gat, O.; Sonenshein, A.L.; Shoham, Y. The Glucuronic Acid Utilization Gene Cluster from Bacillus stearothermophilus T-6. *J. Bacteriol.* **1999**, *181*, 3695–3704, doi:10.1128/JB.181.12.3695-3704.1999.

3. Shulami, S.; Zaide, G.; Zolotnitsky, G.; Langut, Y.; Feld, G.; Sonenshein, A.L.; Shoham, Y. A Two-Component System Regulates the Expression of an ABC Transporter for Xylo-Oligosaccharides in *Geobacillus stearothermophilus*. *Appl. Environ. Microbiol.* **2007**, *73*, 874–884, doi:10.1128/AEM.02367-06.

4. Alalouf, O.; Balazs, Y.; Volkinshtein, M.; Grimpel, Y.; Shoham, G.; Shoham, Y. A New Family of Carbohydrate Esterases Is Represented by a GDSL Hydrolase/Acetylxylan Esterase from *Geobacillus stearothermophilus*. *J. Biol. Chem.* **2011**, *286*, 41993–42001, doi:10.1074/jbc.M111.301051.

5. Shulami, S.; Shenker, O.; Langut, Y.; Lavid, N.; Gat, O.; Zaide, G.; Zehavi, A.; Sonenshein, A.L.; Shoham, Y. Multiple Regulatory Mechanisms Control the Expression of the Geobacillus

stearothermophilus Gene for Extracellular Xylanase. *J. Biol. Chem.* **2014**, *289*, 25957–25975, doi:10.1074/jbc.M114.592873.

6. Daas, M.J.A.; van de Weijer, A.H.P.; de Vos, W.M.; van der Oost, J.; van Kranenburg, R. Isolation of a genetically accessible thermophilic xylan degrading bacterium from compost. *Biotechnol. Biofuels* **2016**, *9*, 210, doi:10.1186/s13068-016-0618-7.

7. Reed, C.J.; Hutinet, G.; de Crécy-Lagard, V. Comparative Genomic Analysis of the DUF34 Protein Family Suggests Role as a Metal Ion Chaperone or Insertase. *Biomolecules* **2021**, *11*, 1282, doi:10.3390/biom11091282.

8. Kanesaki, Y.; Ogura, M. RNA-seq analysis identified glucose-responsive genes and YqfO as a global regulator in Bacillus subtilis. *BMC Res. Notes* **2021**, *14*, 450, doi:10.1186/s13104-021-05869-1.
